# Effect of primaquine dose on the risk of recurrence in patients with uncomplicated *Plasmodium vivax*: a systematic review and individual patient data meta-analysis

**DOI:** 10.1016/S1473-3099(23)00430-9

**Published:** 2024-02

**Authors:** Robert J Commons, Megha Rajasekhar, Peta Edler, Tesfay Abreha, Ghulam R Awab, J Kevin Baird, Bridget E Barber, Cindy S Chu, Liwang Cui, André Daher, Lilia Gonzalez-Ceron, Matthew J Grigg, Jimee Hwang, Harin Karunajeewa, Marcus V G Lacerda, Simone Ladeia-Andrade, Kartini Lidia, Alejandro Llanos-Cuentas, Rhea J Longley, Dhelio B Pereira, Ayodhia P Pasaribu, Sasithon Pukrittayakamee, Komal R Rijal, Inge Sutanto, Walter R J Taylor, Pham V Thanh, Kamala Thriemer, José Luiz F Vieira, James A Watson, Lina M Zuluaga-Idarraga, Nicholas J White, Philippe J Guerin, Julie A Simpson, Ric N Price, WorldWide Antimalarial Resistance Network (WWARN) Vivax Primaquine Dosing Efficacy, Tolerability and Safety Study Group, Bipin Adhikari, Nicholas M Anstey, Ashenafi Assefa, Sarah C Boyd, Nguyen Hoang Chau, Nicholas PJ Day, Tamiru Shibiru Degaga, Arjen M Dondorp, Annette Erhart, Marcelo Urbano Ferreira, Prakash Ghimire, Justin A Green, Gavin CKW Koh, Asrat Hailu Mekuria, Ivo Mueller, Mohammad Nader Naadim, Erni J Nelwan, Francois Nosten, David J Price, Jetsumon Sattabongkot, Kasia Stepniewska, Lorenz von Seidlein, Timothy William, Charles J Woodrow, Adugna Woyessa

**Affiliations:** a Global Health Division, Menzies School of Health Research and Charles Darwin University, Darwin, NT, Australia; b WorldWide Antimalarial Resistance Network (WWARN), Asia-Pacific Regional Centre, Melbourne, VIC, Australia; c General and Subspecialty Medicine, Grampians Health—Ballarat, Ballarat, VIC, Australia; d Centre for Epidemiology and Biostatistics, Melbourne School of Population and Global Health, The University of Melbourne, Melbourne, VIC, Australia; e Department of Medical Biology, The University of Melbourne, Melbourne, VIC, Australia; f Department of Infectious Diseases, The University of Melbourne at the Peter Doherty Institute for Infection and Immunity, Melbourne, VIC, Australia; g ICAP, Columbia University Mailman School of Public Health, Addis Ababa, Ethiopia; h Mahidol Oxford Tropical Medicine Research Unit (MORU), Faculty of Tropical Medicine, Mahidol University, Bangkok, Thailand; i Mahidol Vivax Research Unit, Faculty of Tropical Medicine, Mahidol University, Bangkok, Thailand; j Department of Clinical Tropical Medicine, Faculty of Tropical Medicine, Mahidol University, Bangkok, Thailand; k Nangarhar Medical Faculty, Nangarhar University, Jalalabad, Afghanistan; l Oxford University Clinical Research Unit Indonesia, Faculty of Medicine Universitas Indonesia, Jakarta, Indonesia; m Centre for Tropical Medicine and Global Health, Nuffield Department of Medicine, University of Oxford, Oxford, UK; n QIMR Berghofer Medical Research Institute, Brisbane, QLD, Australia; o Infectious Diseases Society Sabah-Menzies School of Health Research Clinical Research Unit, Kota Kinabalu, Malaysia; p Shoklo Malaria Research Unit, MORU, Faculty of Tropical Medicine, Mahidol University, Mae Sot, Thailand; q Department of Internal Medicine, Morsani College of Medicine, University of South Florida, Tampa, FL, USA; r Fiocruz Clinical Research Platform and Vice‑presidency of Research and Biological Collections, Oswaldo Cruz Foundation (Fiocruz), Rio de Janeiro, Brazil; s Laboratory of Parasitic Diseases, Oswaldo Cruz Foundation (Fiocruz), Rio de Janeiro, Brazil; t Regional Centre for Public Health Research, National Institute for Public Health, Tapachula, Mexico; u US President's Malaria Initiative, Malaria Branch, US Centers for Disease Control and Prevention, Atlanta, GA, USA; v Institute for Global Health Sciences, University of California San Francisco, San Francisco, CA, USA; w Department of Medicine-Western Health, Melbourne Medical School, The University of Melbourne, St Albans, VIC, Australia; x Fundação de Medicina Tropical Dr Heitor Vieira Dourado, Manaus, Brazil; y Instituto Leônidas e Maria Deane, Fiocruz, Manaus, Brazil; z University of Texas Medical Branch, Galveston, TX, USA; aa Global Health and Tropical Medicine, Institute of Hygiene and Tropical Medicine, NOVA University of Lisbon, Lisbon, Portugal; ab Department of Pharmacology and Therapy, Faculty of Medicine and Veterinary Medicine, Universitas Nusa Cendana, Kupang, Indonesia; ac Unit of Leishmaniasis and Malaria, Instituto de Medicina Tropical Alexander von Humboldt, Universidad Peruana Cayetano Heredia, Lima, Peru; ad Population Health and Immunity Division, Walter and Eliza Hall Institute of Medical Research, Melbourne, VIC, Australia; ae Centro de Pesquisa em Medicina Tropical de Rondônia (CEPEM), Porto Velho, Brazil; af Fundação Universidade Federal de Rondônia (UNIR), Porto Velho, Brazil; ag Department of Pediatrics, Medical Faculty, Universitas Sumatera Utara, Medan, Indonesia; ah Central Department of Microbiology, Tribhuvan University, Kirtipur, Nepal; ai Department of Parasitology, Faculty of Medicine, University of Indonesia, Jakarta, Indonesia; aj National Institute of Malariology, Parasitology and Entomology, Hanoi, Viet Nam; ak Federal University of Pará (Universidade Federal do Pará - UFPA), Belém, Brazil; al Oxford University Clinical Research Unit, Hospital for Tropical Diseases, Ho Chi Minh City, Viet Nam; am WWARN, Oxford, UK; an Grupo Malaria, Facultad de Medicina, Universidad de Antioquia, Medellín, Colombia; ao Facultad Nacional de Salud Publica, Universidad de Antioquia, Medellín, Colombia; ap Infectious Diseases Data Observatory (IDDO), Oxford, UK

## Abstract

**Background:**

Primaquine is used to eliminate *Plasmodium vivax* hypnozoites, but its optimal dosing regimen remains unclear. We undertook a systematic review and individual patient data meta-analysis to investigate the efficacy and tolerability of different primaquine dosing regimens to prevent *P vivax* recurrence.

**Methods:**

For this systematic review and individual patient data meta-analysis, we searched MEDLINE, Web of Science, Embase, and Cochrane Central for prospective clinical studies of uncomplicated *P vivax* from endemic countries published between Jan 1, 2000, and June 8, 2023. We included studies if they had active follow-up of at least 28 days, and if they included a treatment group with daily primaquine given over multiple days, where primaquine was commenced within 7 days of schizontocidal treatment and was given alone or coadministered with chloroquine or one of four artemisinin-based combination therapies (ie, artemether–lumefantrine, artesunate–mefloquine, artesunate–amodiaquine, or dihydroartemisinin–piperaquine). We excluded studies if they were on prevention, prophylaxis, or patients with severe malaria, or if data were extracted retrospectively from medical records outside of a planned trial. For the meta-analysis, we contacted the investigators of eligible trials to request individual patient data and we then pooled data that were made available by Aug 23, 2021. We assessed the effects of total dose and duration of primaquine regimens on the rate of first *P vivax* recurrence between day 7 and day 180 by Cox's proportional hazards regression (efficacy analysis). The effect of primaquine daily dose on gastrointestinal symptoms on days 5–7 was assessed by modified Poisson regression (tolerability analysis). The study was registered with PROSPERO, CRD42019154470.

**Findings:**

Of 226 identified studies, 23 studies with patient-level data from 6879 patients from 16 countries were included in the efficacy analysis. At day 180, the risk of recurrence was 51·0% (95% CI 48·2–53·9) in 1470 patients treated without primaquine, 19·3% (16·9–21·9) in 2569 patients treated with a low total dose of primaquine (approximately 3·5 mg/kg), and 8·1% (7·0–9·4) in 2811 patients treated with a high total dose of primaquine (approximately 7 mg/kg), regardless of primaquine treatment duration. Compared with treatment without primaquine, the rate of *P vivax* recurrence was lower after treatment with low-dose primaquine (adjusted hazard ratio 0·21, 95% CI 0·17–0·27; p<0·0001) and high-dose primaquine (0·10, 0·08–0·12; p<0·0001). High-dose primaquine had greater efficacy than low-dose primaquine in regions with high and low relapse periodicity (ie, the time from initial infection to vivax relapse). 16 studies with patient-level data from 5609 patients from ten countries were included in the tolerability analysis. Gastrointestinal symptoms on days 5–7 were reported by 4·0% (95% CI 0·0–8·7) of 893 patients treated without primaquine, 6·2% (0·5–12·0) of 737 patients treated with a low daily dose of primaquine (approximately 0·25 mg/kg per day), 5·9% (1·8–10·1) of 1123 patients treated with an intermediate daily dose (approximately 0·5 mg/kg per day) and 10·9% (5·7–16·1) of 1178 patients treated with a high daily dose (approximately 1 mg/kg per day). 20 of 23 studies included in the efficacy analysis and 15 of 16 in the tolerability analysis had a low or unclear risk of bias.

**Interpretation:**

Increasing the total dose of primaquine from 3·5 mg/kg to 7 mg/kg can reduce *P vivax* recurrences by more than 50% in most endemic regions, with a small associated increase in gastrointestinal symptoms.

**Funding:**

Australian National Health and Medical Research Council, Bill & Melinda Gates Foundation, and Medicines for Malaria Venture.

## Introduction

*Plasmodium vivax* causes between 4·9 million and 14·3 million cases of malaria each year, with 2·5 billion people at risk globally.^1^, ^2^ An estimated 66–95% of these malaria cases are relapses, arising from reactivation of liver-stage hypnozoites.^3^ The 8-aminoquinolines—primaquine and tafenoquine—are the only available antimalarials that eliminate hypnozoites and thus prevent relapses, but they can cause severe haemolysis in individuals with glucose-6-phosphate dehydrogenase (G6PD) deficiency.^4^

Primaquine has been the mainstay of anti-relapse treatment for *P vivax* for almost 70 years; however, the optimal dose remains unclear. Despite concerns of severe drug-induced haemolysis in at-risk patients, point-of-care G6PD testing is often unavailable in malaria-endemic areas and most national malaria programmes recommend low total dose primaquine (3·5 mg/kg) administered during 14 days to reduce the risk of haemolysis.^4^ WHO recommends a higher total dose (7 mg/kg) in east Asia and Oceania,^5^ but this dose is used predominantly in well resourced, non-endemic countries where patients have greater access to routine G6PD testing.

In the past decade, advances in point-of-care G6PD tests have reinvigorated calls for optimising the dose of primaquine in individuals without G6PD deficiency. Only a few clinical trials have compared low-dose (3·5 mg/kg total) with high-dose (7 mg/kg total) primaquine, and these studies have generally had small sample sizes and short durations of follow-up.^6^, ^7^, ^8^, ^9^, ^10^, ^11^ We did a systematic review and individual patient data meta-analysis to explore the effect of primaquine dose on the risk of *P vivax* recurrences to identify the most efficacious doses of primaquine across different endemic settings and to assess their tolerability.

## Methods

### Search strategy and selection criteria

For this systematic review and individual patient data meta-analysis, we searched MEDLINE, Web of Science, Embase, and Cochrane Central for clinical antimalarial efficacy studies of patients with uncomplicated *P vivax* monoinfections in any language published between Jan 1, 2000, and Aug 23, 2021, and we updated the search in June, 2023, to include studies published until June 8, 2023. The review was undertaken according to the PRISMA individual patient data statement (appendix pp 3–6) with search terms as described previously (appendix p 7).^12^ We also reviewed the studies in the reference lists of the identified articles. We included studies if they were prospective with active follow-up of at least 28 days, if they included a treatment group with daily primaquine given over multiple days, where primaquine was commenced within 7 days of schizontocidal treatment, and if primaquine was given alone or coadministered with chloroquine or one of four artemisinin-based combination therapies (ie, artemether–lumefantrine, artesunate–mefloquine, artesunate–amodiaquine, or dihydroartemisinin–piperaquine). Reviews and animal studies; studies on prevention, prophylaxis, or patients with severe malaria; and studies where schizontocidal treatment was unsupervised or where data were extracted retrospectively from medical records outside of a planned trial were excluded. In June, 2023, we also did a post-hoc systematic review of the Scopus database, using the same search strategy and selection criteria. The systematic review was undertaken by two reviewers (RJC and RNP), with discrepancies resolved by discussion. The protocol was registered with PROSPERO, CRD42019154470.

**Research in context**

**Evidence before this study**

We identified randomised controlled trials comparing primaquine regimens with low and high total doses for *Plasmodium vivax* malaria, published in any language, from Jan 1, 1960, to June 8, 2023, using the terms “vivax” and “primaquine” in MEDLINE, Web of Science, Embase, and Cochrane Central. We identified six trials, of which only two reported high-dose primaquine (7 mg/kg) to be more efficacious at preventing recurrences than low-dose primaquine (3·5 mg/kg). However, study designs were generally confounded by short durations of follow-up and small sample sizes.

**Added value of this study**

This individual patient data meta-analysis included data from 26 studies done in 16 countries (across both efficacy and tolerability analyses) and, to date, represents the largest meta-analysis assessing the efficacy and tolerability of primaquine regimens for radical cure of *P vivax* malaria. The results show the benefit of high total dose (7 mg/kg) compared with low total dose (3·5 mg/kg) primaquine to reduce vivax recurrences across most vivax-endemic regions. The increase in the daily dose of primaquine (from 0·25 mg/kg per day to 0·5–1 mg/kg per day) resulted in a small increase in gastrointestinal adverse events. These results are co-reported with a meta-analysis of the risk of haemolysis with different primaquine daily doses.

**Implications of all the available evidence**

High total dose primaquine (7 mg/kg) might halve the risk of *P vivax* recurrences in most vivax-endemic regions over 180 days of follow-up. Increased daily doses need to be balanced against increased risk of gastrointestinal side-effects and haemolysis in vulnerable patients.

### Data collation

Investigators of eligible studies were invited to share individual patient data, and also to contribute data from unpublished studies they had been involved with, by Aug 23, 2021. We did not request individual patient data for studies published between Aug 24, 2021, and June 8, 2023, due to the long time required to obtain approval and collate these data. Shared data were de-identified and collated using a standardised methodology in the WorldWide Antimalarial Resistance Network repository.^13^ We excluded individual patient data if data on age, sex, baseline parasite density, or total mg/kg dose of primaquine given were missing. Patients with severe malaria, with mixed-species infection at enrolment, who were pregnant, who had received adjunctive antimalarials within 14 days of starting treatment, or who were treated with intermittent primaquine regimens were also excluded.

Shared data were obtained according to ethical approvals from the country of origin and original study. The data were anonymised and unable to be linked to individuals. As such, our analysis did not require additional ethical approval according to the guidelines of the Oxford Central University Research Ethics Committee.

### Outcomes

The primary efficacy outcome was any *P vivax* recurrence between day 7 and day 180. The secondary efficacy outcomes were any *P vivax* recurrence between day 7 and day 365 and symptomatic *P vivax* recurrences between day 7 and day 365. The primary tolerability outcome was the reported incidence of gastrointestinal disturbance on days 5–7, a composite endpoint defined as either vomiting, anorexia, or diarrhoea, and was assessed in all patients as well as in children (aged <15 years) and adults (aged ≥15 years) separately. This timepoint was selected to assess primaquine-related symptoms after acute malaria symptoms had resolved and concurrent schizontocidal treatment had been completed. Secondary tolerability outcomes were reported incidence of the composite outcome on days 1–2 during acute malaria (with all patients having started primaquine treatment on day 0); reported incidence of vomiting, nausea, anorexia, abdominal pain, diarrhoea, or dizziness on days 5–7 as separate outcomes; and vomiting within 1 h of primaquine administration (ie, acute vomiting).

### Data analysis

The duration of primaquine treatment was categorised as 7 days or 14 days and was explored in treatment groups with similar total doses. Primaquine dosing supervision was defined as unsupervised (fewer than two observed doses), partly supervised (two or more observed doses but not all doses), and fully supervised (all doses observed). In the efficacy analyses, which were restricted to studies with a minimum of 42 days of follow-up (appendix p 8), total primaquine dose was categorised into very low dose (<2 mg base per kg), low dose (2 mg base per kg to <5 mg base per kg, reflecting a target dose of 3·5 mg base per kg), and high dose (≥5 mg base per kg, reflecting a target dose of 7 mg base per kg). In an analysis to measure the effect of small changes in dose, the total dose was treated as a continuous variable. The daily primaquine dose was used for tolerability analyses, which were restricted to studies with symptom checklist data and patients treated with primaquine commencing within 3 days of starting schizontocidal treatment (appendix p 8). The daily dose was categorised as low dose (<0·375 mg base per kg per day), intermediate dose (≥0·375 mg base per kg per day to <0·75 mg base per kg per day), and high dose (≥0·75 mg base per kg per day), reflecting the spread of dosing around the two recommended daily dosing regimens (0·25 mg base per kg per day and 0·5 mg base per kg per day) and a proposed higher daily dosing regimen (1 mg base per kg per day).^14^ Age was categorised into three groups (<5 years, 5 years to <15 years, and ≥15 years) for the primary efficacy and primary gastrointestinal outcomes; if the number of patients younger than 5 years was small, age was categorised into two groups (<15 years and ≥15 years). Separate analyses of the primary efficacy outcome were subgrouped by relapse periodicity (ie, the time from initial infection to vivax relapse), which was classified as high or low by geographical region, with high-periodicity regions defined as having a median relapse periodicity of 47 days or less and low-periodicity regions as having a median relapse periodicity of more than 47 days.^15^ Additional procedures are described in the appendix (p 8).

The risks of recurrence between day 7 and day 180 (and between day 7 and day 365) were calculated by Kaplan-Meier survival analyses stratified by primaquine treatment group (appendix p 8). The associations between (1) primaquine total dose or (2) target primaquine duration and the time to the first vivax recurrence between day 7 and day 180 were estimated separately by Cox's proportional hazards regression, with the proportional hazards assumption checked visually. A natural cubic spline model with four knots was used to investigate the relationship between the continuous total dose of primaquine and the risk of first vivax recurrence between day 7 and day 180. The knots were set at the fifth (3·0 mg/kg), 35th (3·8 mg/kg), 65th (6·8 mg/kg), and 95th (8·6 mg/kg) percentiles of the primaquine total dose data. Cox models, including the model underlying the spline model, were adjusted for age, sex, and log_10_ baseline parasite density, with shared frailty for study site, on the basis of a directed acyclic graph (appendix p 9).

Incidence rates of multiple recurrent episodes of *P vivax* parasitaemia between day 7 and day 180 (and between day 7 and day 365) were calculated from studies with a minimum 180 days of follow-up that followed up patients through multiple episodes of vivax parasitaemia (appendix p 8).

The association between the daily primaquine dose and the composite gastrointestinal endpoint on days 5–7 was assessed using a generalised estimating equation Poisson model with robust standard error estimates, adjusting for age, sex, and log_10_ baseline parasite density, with effect modification by age category, exchangeable correlation structure, and clustering by study site (appendix p 8).

Risk of bias assessments including assessment for within study bias, inclusion bias, and between study heterogeneity are described in the appendix (p 8). Assessment of publication bias was not done because the objective of the original studies did not align with our primary research question. Analyses were undertaken in R (version 4.1.3) and Stata (version 17) according to an a-priori statistical analysis plan.^16^

### Role of the funding source

The funders of the study had no role in study design, data collection, data analysis, data interpretation, or writing of the report.

## Results

**Figure 1:**
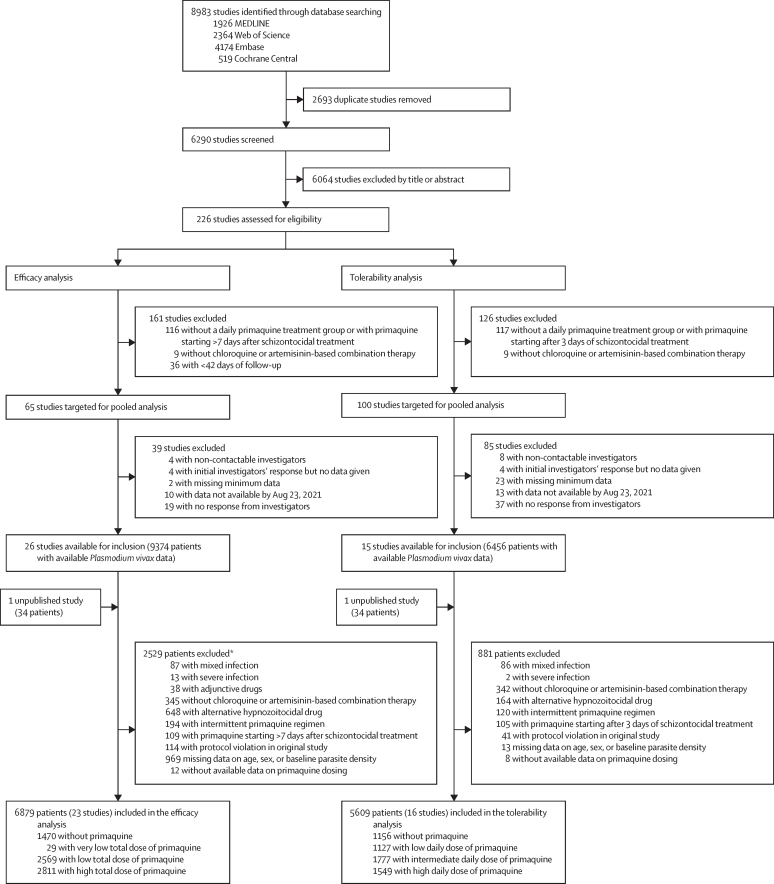
Study selection *Includes all patients from four studies excluded on patient-level factors (appendix p 13) and individual patients from additional studies.

Between Jan 1, 2000, and June 8, 2023, we identified 8983 studies via database searching (figure 1). After removing 2693 duplicates and excluding 6064 studies in the title and abstract screening, we assessed 226 *P vivax* efficacy studies for eligibility, of which 65 met the inclusion criteria for the efficacy analysis. Investigators from 26 (40·0%) studies shared individual patient data on 9374 patients. An additional unpublished study with data on 34 patients was shared. 2529 patients were excluded, resulting in 6879 patients from 23 studies (one unpublished) and 16 countries in the final analysis (figure 1; appendix pp 10–17).^14^, ^17^, ^18^, ^19^, ^20^, ^21^, ^22^, ^23^, ^24^, ^25^, ^26^, ^27^, ^28^, ^29^, ^30^, ^31^, ^32^, ^33^, ^34^, ^35^, ^36^, ^37^ In the post-hoc systematic review in Scopus, we did not identify additional eligible studies.

The median age of patients in the efficacy analysis was 18·0 years (IQR 10·0–30·0), with 467 (6·8%) of 6879 patients younger than 5 years (table). Overall, 4257 (61·9%) patients were male, 2622 (38·1%) were female, 4938 (71·8%) were from the Asia-Pacific region, 995 (14·5%) from Africa, and 946 (13·8%) from the Americas. 5684 (82·6%) patients in 16 studies were followed up for 180 days or more,^14^, ^19^, ^20^, ^21^, ^23^, ^25^, ^26^, ^27^, ^28^, ^29^, ^31^, ^32^, ^34^, ^35^, ^36^, ^37^ and 4620 (67·2%) patients received schizontocidal treatment with chloroquine (table).

**Table:** Demographic and baseline characteristics

	**Overall (n=6879)**	**Primaquine dose**			
		No primaquine (n=1470)	Very low total dose (<2 mg/kg; n=29)	Low total dose (2 mg/kg to <5 mg/kg; n=2569)	High total dose (≥5 mg/kg; n=2811)
**Sex**					
Male	4257 (61·9%)	927 (63·1%)	21 (72·4%)	1526 (59·4%)	1783 (63·4%)
Female	2622 (38·1%)	543 (36·9%)	8 (27·6%)	1043 (40·6%)	1028 (36·6%)
**Age**					
Median, years	18·0 (10·0–30·0)	19·0 (11·6–30·0)	19·4 (10·0–23·0)	19·0 (10·0–35·0)	17·0 (10·9–27·8)
<5 years	467 (6·8%)	89 (6·1%)	1 (3·4%)	191 (7·4%)	186 (6·6%)
5 years to <15 years	2200 (32·0%)	404 (27·5%)	9 (31·0%)	827 (32·2%)	960 (34·2%)
≥15 years	4212 (61·2%)	977 (66·5%)	19 (65·5%)	1551 (60·4%)	1665 (59·2%)
**Enrolment variables**					
Weight, kg	50·0 (30·0–60·0)	50·0 (31·1–60·0)	56·0 (37·3–67·0)	55·0 (37·5–66·1)	46·0 (26·5–55·2)
Malnutrition*	101/485 (20·8%)	26/106 (24·5%)	0/2	28/151 (18·5%)	47/226 (20·8%)
Presence or recent history of fever	5774/6309 (91·5%)	1349/1469 (91·8%)	23/29 (79·3%)	1866/2056 (90·8%)	2536/2755 (92·1%)
Parasitaemia, parasites per mL	3400·0 (1118·0–8866·7)	4387·0 (1700·0–10800·0)	2777·8 (740·7–5814·8)	2680·0 (960·0–5920·0)	3936·0 (1018·5–11711·1)
Haemoglobin, g/dL†	12·6 (1·8)	12·6 (1·8)	12·6 (2·1)	12·5 (1·9)	12·7 (1·8)
**Schizontocidal treatment**					
Chloroquine	4620 (67·2%)	1139 (77·5%)	24 (82·8%)	1872 (72·9%)	1585 (56·4%)
Artemether–lumefantrine	353 (5·1%)	135 (9·2%)	0	185 (7·2%)	33 (1·2%)
Artesunate–amodiaquine	229 (3·3%)	0	0	197 (7·7%)	32 (1·1%)
Artesunate–mefloquine	90 (1·3%)	0	1 (3·4%)	89 (3·5%)	0
Dihydroartemisinin–piperaquine	1587 (23·1%)	196 (13·3%)	4 (13·8%)	226 (8·8%)	1161 (41·3%)
**Primaquine dosing**					
Total dose, mg/kg	5·4 (3·3–7·2)	..	1·0 (0·8–1·5)	3·3 (3·0–3·9)	7·2 (6·7–7·9)
Start day	0·0 (0·0–0·0)	..	0·0 (0·0–0·0)	0·0 (0·0–0·0)	0·0 (0·0–0·0)
**Primaquine duration**					
7 days	2302/5373 (42·8%)	..	13/25 (52·0%)	1076/2566 (41·9%)	1213/2782 (43·6%)
14 days	3071/5373 (57·2%)	..	12/25 (48·0%)	1490/2566 (58·1%)	1569/2782 (56·4%)
**Primaquine dose derived from**					
Actual dosing	3175/5409 (58·7%)	..	28 (96·6%)	431 (16·8%)	2716 (96·6%)
Protocol dosing	2234/5409 (41·3%)	..	1 (3·4%)	2138 (83·2%)	95 (3·4%)
**Primaquine dosing supervision**					
Unsupervised	151/5409 (2·8%)	..	4 (13·8%)	117 (4·6%)	30 (1·1%)
Partly supervised	1803/5409 (33·3%)	..	3 (10·3%)	1780 (69·3%)	20 (0·7%)
Fully supervised	3455/5409 (63·9%)	..	22 (75·9%)	672 (26·2%)	2761 (98·2%)
**Primaquine administered with food**					
No	423/5409 (7·8%)	..	5 (17·2%)	211 (8·2%)	207 (7·4%)
Yes	2840/5409 (52·5%)	..	14 (48·3%)	962 (37·4%)	1864 (66·3%)
Recommended	2146/5409 (39·7%)	..	10 (34·5%)	1396 (54·3%)	740 (26·3%)
**Study follow-up**					
<180 days	1195 (17·4%)	27 (1·8%)	2 (6·9%)	1123 (43·7%)	43 (1·5%)
≥180 days	5684 (82·6%)	1443 (98·2%)	27 (93·1%)	1446 (56·3%)	2768 (98·5%)
**Relapse periodicity**					
Low	2927 (42·5%)	808 (55·0%)	18 (62·1%)	1310 (51·0%)	791 (28·1%)
High	3952 (57·5%)	662 (45·0%)	11 (37·9%)	1259 (49·0%)	2020 (71·9%)
**Transmission intensity**‡					
Low	817 (11·9%)	224 (15·2%)	6 (20·7%)	230 (9·0%)	357 (12·7%)
Moderate	3253 (47·3%)	439 (29·9%)	7 (24·1%)	1146 (44·6%)	1661 (59·1%)
High	2809 (40·8%)	807 (54·9%)	16 (55·2%)	1193 (46·4%)	793 (28·2%)
**Region**					
Africa	995 (14·5%)	328 (22·3%)	8 (27·6%)	202 (7·9%)	457 (16·3%)
Americas	946 (13·8%)	119 (8·1%)	3 (10·3%)	805 (31·3%)	19 (0·7%)
Asia-Pacific	4938 (71·8%)	1023 (69·6%)	18 (62·1%)	1562 (60·8%)	2335 (83·1%)
**G6PD status**§					
<30% activity	46/5327 (0·9%)	30/1455 (2·1%)	0/26	14/1132 (1·2%)	2/2714 (0·1%)
≥30% activity	5281/5327 (99·1%)	1425/1455 (97·9%)	26/26 (100%)	1118/1132 (98·8%)	2712/2714 (99·9%)

Data are n (%), median (IQR), mean (SD), or n/N (%). Data were not available for 647 patients on weight and 825 patients on baseline haemoglobin concentration. G6PD=glucose-6-phosphate dehydrogenase.

* The nutritional status of children younger than 5 years was calculated as a weight-for-age Z score, using the igrowup package developed by WHO,^38^ with children with Z scores smaller than −2 classified as having malnutrition and malnutrition status considered missing if Z scores were smaller than −6 or larger than 6.

† If haemoglobin was not measured, haematocrit was converted to haemoglobin using the formula: haemoglobin (g/dL)=(haematocrit [%] – 5·62)/2·60.

‡ Transmission intensity of study sites was classified as low (<1 case per 1000 person-years), moderate (1 case to <10 cases per 1000 person-years), and high (≥10 cases per 1000 person-years) on the basis of subnational malaria incidence estimates for the median year of enrolment.^1^

§ G6PD deficiency was categorised as deficient (<30% activity or an abnormal qualitative test) and normal (≥30% activity or a normal qualitative test).

1470 (21·4%) patients were treated without primaquine, 29 (0·4%) were treated with a very low total dose of primaquine, 2569 (37·3%) with a low total dose, and 2811 (40·9%) with a high total dose (figure 1; appendix pp 18–21). Of 5409 patients treated with primaquine, dosing was fully supervised in 3455 (63·9%) patients; in 3175 (58·7%) patients, mg/kg dosing was calculated from the actual administered dose.

Compared with other treatment groups, patients treated with high-dose primaquine were more likely to come from regions with high relapse periodicity, have their primaquine dosing fully supervised, have their dose derived from their actual administered dose, and receive dihydroartemisinin–piperaquine as a schizontocidal agent (table). Studies and patients that were eligible but not included in the efficacy analysis had similar baseline characteristics to included studies and patients, although included studies recruited patients more recently and had a higher proportion of female patients (appendix p 22). Of the 23 included studies, 15 (one unpublished; 65·2%) were randomised controlled trials,^14^, ^17^, ^19^, ^20^, ^23^, ^27^, ^28^, ^29^, ^30^, ^32^, ^34^, ^35^, ^36^, ^37^ and eight (34·8%) were non-randomised clinical efficacy studies,^18^, ^21^, ^22^, ^24^, ^25^, ^26^, ^31^, ^33^ with 20 (one unpublished; 87·0%) having low or unclear risk of bias (appendix pp 23–24).^14^, ^17^, ^18^, ^19^, ^20^, ^23^, ^24^, ^25^, ^26^, ^27^, ^28^, ^29^, ^30^, ^32^, ^33^, ^34^, ^35^, ^36^, ^37^ Because 24 (82·8%) of 29 patients who were administered less than 2 mg/kg of primaquine stopped their treatment early, the very low total dose treatment category was not included in subsequent analyses.

**Figure 2:**
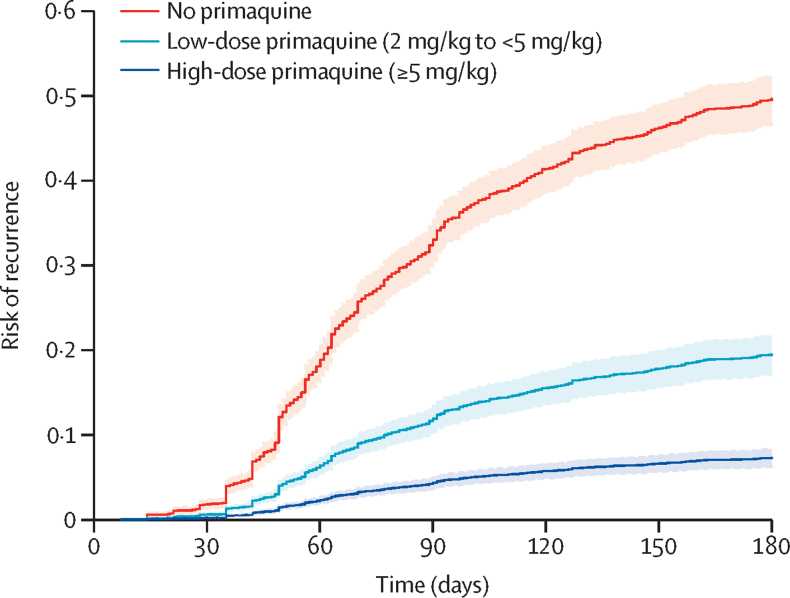
Risk of first *Plasmodium vivax* recurrence in patients between day 7 and day 180 Shaded regions show 95% CIs. Estimates derived from a Cox regression model adjusted for age, sex, and log_10_ baseline parasite density. Relapse periodicity was not included in the model because the proportional hazards assumption was expected to be violated for this variable, which is defined by the time to recurrence. Risk of recurrence assumes covariates at mean values and zero effect from study site.

**Figure 3:**
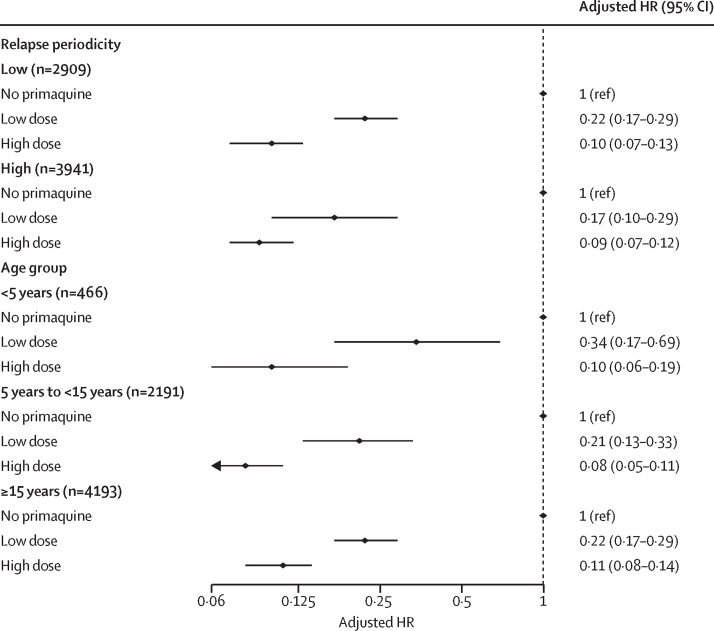
Hazard ratio for first *Plasmodium vivax* recurrence between day 7 and day 180 with high total dose or low total dose of primaquine compared with no primaquine, subgrouped by relapse periodicity and age group Estimates derived from the Cox regression models were adjusted for age, sex, and log_10_ baseline parasite density, with shared frailty for study site. HR=hazard ratio.

The cumulative risk of recurrence at 180 days was 51·0% (95% CI 48·2–53·9; 628 recurrences) in patients treated without primaquine, 19·3% (16·9–21·9; 234 recurrences) in those treated with low-dose primaquine, and 8·1% (7·0–9·4; 183 recurrences) in those treated with high-dose primaquine (appendix p 25). Patients treated with either low-dose primaquine (adjusted hazard ratio [HR] 0·21, 95% CI 0·17–0·27; p<0·0001) or high-dose primaquine (0·10, 0·08–0·12; p<0·0001) had a reduced rate of first vivax recurrence between day 7 and day 180 compared with those treated without primaquine (appendix p 26). The rate of recurrence was lower after treatment with high-dose primaquine than after treatment with low-dose primaquine (0·45, 0·34–0·60; p<0·0001; figure 2). Estimated adjusted HRs were similar in multiple sensitivity analyses, including restriction to eight studies (one unpublished) that randomly assigned patients to primaquine versus no primaquine,^14^, ^19^, ^27^, ^28^, ^29^, ^34^, ^37^ and 11 studies (one unpublished) in which primaquine was fully supervised (appendix pp 27–28).^14^, ^20^, ^21^, ^22^, ^23^, ^25^, ^26^, ^29^, ^32^, ^35^ In subgroup analyses, compared with patients not treated with primaquine, low-dose primaquine was associated with lower rates of first vivax recurrence in patients younger than 5 years (adjusted HR 0·34, 95% CI 0·17–0·69), in those aged 5 years or older but younger than 15 years (0·21, 0·13–0·33), and in those aged 15 years or older (0·22, 0·17–0·29; figure 3). High-dose primaquine had higher efficacy compared with low-dose primaquine both in regions of high relapse periodicity (adjusted HR 0·55, 95% CI 0·31–0·96; p=0·04) and in regions of low relapse periodicity (0·42, 0·28–0·63; p<0·0001).

At 365 days, the risks of recurrence were 59·6% (56·6–62·7; 688 recurrences) in patients treated without primaquine, 28·2% (24·7–32·0; 266 recurrences) in patients treated with low-dose primaquine, and 17·3% (15·1–19·9; 286 recurrences) in patients treated with high-dose primaquine. In each treatment group, the risk of recurrence varied substantially between studies (appendix pp 29–30).

The target primaquine duration was available for 5348 patients. Of 2566 patients treated with a low total dose of primaquine, 1076 (41·9%) were treated for 7 days and 1490 (58·1%) for 14 days. For patients treated with low-dose primaquine, we did not compare the rate of recurrence from day 7 to day 180 in patients treated for 7 days versus 14 days, because 958 (89·0%) of 1076 patients treated with low-dose primaquine for 7 days were only followed up for 63 days, whereas 1327 (89·1%) of 1490 patients treated for 14 days were followed up for 180 days. Of 2782 patients treated with a high total dose of primaquine, 1213 (43·6%) were treated for 7 days and 1569 (56·4%) for 14 days. In patients treated with high-dose primaquine, we found no difference in the rate of first *P vivax* recurrence with 14-day versus 7-day treatment (adjusted HR 0·80, 0·59–1·09; p=0·15) and HRs were similar in sensitivity analyses (appendix pp 31–32).

**Figure 4:**
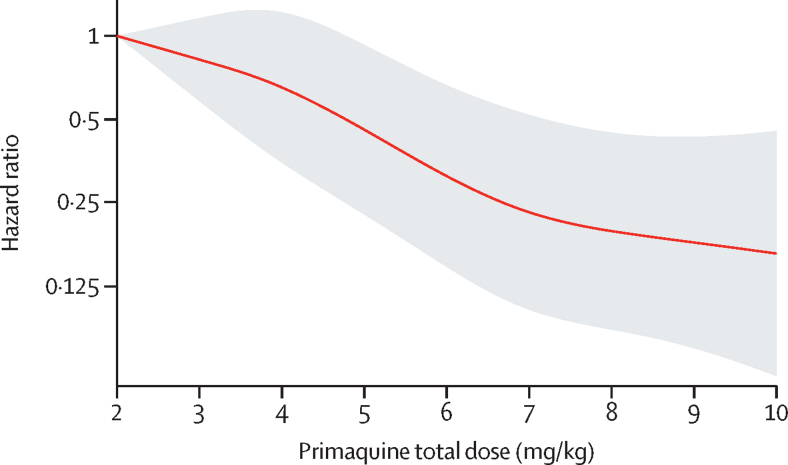
Relative hazard of first *Plasmodium vivax* recurrence between day 7 and day 180 associated with primaquine total mg/kg dose The reference value was set at 2 mg/kg. Shaded region shows 95% CIs. Spline model was based on Cox regression model adjusted for age, sex, and log_10_ baseline parasite density, with shared frailty for study site. Age, sex, and log_10_ baseline parasite density were set at their mean values.

In patients treated with primaquine, a 1 mg/kg increase in the total primaquine dose from a reference of 2 mg/kg was associated with an adjusted HR of 0·82 (95% CI 0·73–0·91; p=0·0004). There was a markedly slower rate of decrease in the HR when the total dose exceeded 6–7 mg/kg (figure 4).

In total, 12 studies (5471 patients) followed up patients through multiple episodes of vivax parasitaemia for at least 180 days.^14^, ^19^, ^20^, ^26^, ^27^, ^28^, ^29^, ^32^, ^34^, ^35^, ^36^, ^37^ The incidence rate for all recurrences between day 7 and day 180 was 1·84 recurrences per person-year (95% CI 1·74–1·95) in patients not receiving primaquine, 0·47 recurrences per person-year (0·42–0·52) after treatment with low-dose primaquine, and 0·20 recurrences per person-year (0·18–0·22) after treatment with high-dose primaquine (appendix pp 33–36).

Of the 226 identified studies in the systematic review, 100 met the inclusion criteria for the tolerability analysis. Investigators from 15 studies shared individual patient data on 6456 patients. An additional unpublished study with data on 34 patients was shared. After excluding 881 patients, the tolerability analysis comprised 5609 patients from 16 studies (one unpublished) and ten countries (figure 1; appendix p 10).^10^, ^14^, ^17^, ^20^, ^21^, ^23^, ^26^, ^27^, ^28^, ^29^, ^32^, ^35^, ^36^, ^39^, ^40^ 12 (one unpublished; 75·0%) were randomised controlled trials,^10^, ^14^, ^17^, ^20^, ^23^, ^27^, ^28^, ^29^, ^32^, ^35^, ^36^ and four (25·0%) were non-randomised clinical efficacy studies,^21^, ^26^, ^39^, ^40^ with 15 (one unpublished; 93·8%) having low or unclear risk of bias (appendix pp 23–24).^10^, ^14^, ^17^, ^20^, ^23^, ^26^, ^27^, ^28^, ^29^, ^32^, ^35^, ^36^, ^39^, ^40^ In the post-hoc systematic review in Scopus, we did not identify additional eligible studies. Of 5609 patients, 1156 (20·6%) were not treated with primaquine, 1127 (20·1%) were treated with a low daily dose of primaquine, 1777 (31·7%) with an intermediate daily dose, and 1549 (27·6%) with a high daily dose; 406 (7·2%) were younger than 5 years, 1823 (32·5%) were aged 5 years or older but younger than 15 years, and 3380 (60·3%) were aged 15 years or older. Additional baseline characteristics are shown in the appendix (pp 37–38).

**Figure 5:**
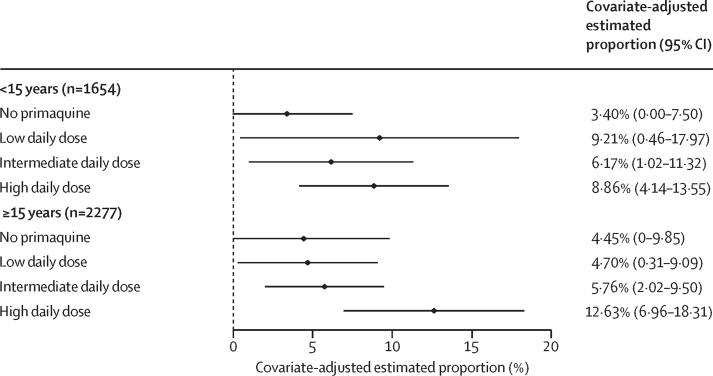
Covariate-adjusted estimated proportion of patients with gastrointestinal symptoms on days 5–7 by primaquine treatment regimen and age group Gastrointestinal symptoms are represented by a composite endpoint indicating presence of vomiting, diarrhoea, or anorexia on days 5–7. A generalised estimating equation Poisson model was fit to data from 3931 patients, and the adjusted proportions were estimated using mean values for age, sex, and log_10_ baseline parasite density. Owing to their low number (n=295 across all dose categories), children younger than 5 years were grouped with children aged 5 years or older but younger than 15 years. Results for each age group are presented in the appendix (p 41).

On days 5–7, the incidence of gastrointestinal symptoms as measured by the composite endpoint was higher in patients treated with primaquine, particularly the high daily dose regimen, than in patients not treated with primaquine (appendix p 39). Across all ages, the higher incidence of the gastrointestinal outcome persisted after adjustment for confounders (4·0% [95% CI 0·0–8·7] in 893 patients treated without primaquine, 6·2% [0·5–12·0] in 737 patients treated with a low daily dose of primaquine, 5·9% [1·8–10·1] in 1123 patients treated with an intermediate daily dose, and 10·9% [5·7–16·1] in 1178 patients treated with a high daily dose). A greater proportion of children younger than 15 years treated with primaquine (all doses) reported gastrointestinal symptoms compared with children treated without primaquine, whereas a greater proportion of patients aged 15 years or older treated with a high daily dose of primaquine reported symptoms compared with patients receiving other doses or no primaquine (figure 5; appendix pp 40–42). Findings were consistent in patients asked about all three symptoms (appendix pp 43–44).

The overall risk of acute vomiting was low, but higher on days 0–2 (1·5% [54/3501]) than on days 3–14 (0·4% [12/3152]). There was no clear association between primaquine dose and risk of acute vomiting (appendix p 45).

When assessed early in the treatment (days 1–2), similar proportions of children younger than 15 years reported gastrointestinal symptoms across all treatment groups after adjusting for confounders, whereas a greater proportion of patients aged 15 years or older treated with the intermediate or high daily dose of primaquine reported symptoms (appendix pp 46–47). Considered separately, the incidence of vomiting, diarrhoea, or anorexia on days 5–7 was higher in children treated with primaquine, and similar across dose groups, than in those not receiving primaquine (appendix p 39). Similarly, compared with adults not treated with primaquine, a greater proportion of adults treated with primaquine had these symptoms, as well as nausea, abdominal pain, or dizziness on days 5–7, particularly after the high daily dose of primaquine compared with the low or intermediate daily dose (appendix p 39).

## Discussion

This individual patient data meta-analysis shows that patients treated with a high total dose of primaquine (approximately 7 mg/kg) are at significantly lower risk of *P vivax* recurrence within 180 days compared with those treated with low-dose primaquine (approximately 3·5 mg/kg); this finding was consistent across both areas with low and high relapse periodicity. Primaquine was reasonably well tolerated at all doses.

Although the dose–response relationship between total primaquine dose and anti-relapse efficacy has long been recognised,^41^ the benefit or otherwise of a dose increase across different regions has remained unclear. WHO guidelines recommend 7 mg/kg in east Asia and Oceania,^5^ supported by increased recurrence rates in some studies with low-dose primaquine.^42^, ^43^ However, in this region, these guidelines have only been implemented by Malaysia.^4^ The hesitance to use high-dose primaquine relates to uncertainty around the risk versus benefit, because higher doses of primaquine are associated with increased risk of haemolysis and gastrointestinal intolerance.^44^ Only six studies have compared low-dose and high-dose primaquine directly,^6^, ^7^, ^8^, ^9^, ^10^, ^11^ including two studies done in India with a follow-up of 6 months,^8^, ^9^ and two studies in Thailand (both of which only followed up patients for 28 days).^7^, ^10^

In our analysis, high-dose primaquine reduced the risk of recurrent *P vivax* by about half compared with low-dose primaquine. The benefit was greatest in children younger than 5 years, in whom low-dose primaquine had lower efficacy (approximately 66%) compared with older age groups (approximately 78%; figure 3). Young children are particularly at risk of malaria and recurrent infections, probably reflecting their lower parasite immunity compared with adults. Paediatric doses of antimalarials are usually extrapolated from adult pharmacokinetic studies, often resulting in underdosing because young children have lower drug exposures for the same mg/kg dose. Previous individual patient data analyses have shown that antimalarials have been registered initially at suboptimal doses in children.^45^, ^46^ Our findings support a previous study done on the Thailand–Myanmar border, which reported that, at day 7, primaquine and carboxyprimaquine plasma concentrations in children younger than 5 years were approximately half of those found in adults aged 30 years after a total dose of 7 mg/kg.^47^ Although our analysis supports the use of high doses of primaquine in children, further studies are warranted to establish whether primaquine doses should be further increased in young children compared with adults. The absence of child-friendly primaquine formulations produced through good manufacturing practice provides an added barrier to primaquine treatment in young children.

Overall, the benefit of high-dose over low-dose primaquine was apparent in regions with both low and high relapse periodicity. When unsupervised, primaquine treatment is poorly adhered to,^48^, ^49^ and even prospective studies with partial supervision have found reduced efficacy.^27^ Thus, the intake of a higher daily dose might lead to an increased total dose of primaquine and adequate efficacy, even in the setting of missed doses. High-dose primaquine might also overcome other factors associated with reduced efficacy, such as some *CYP2D6* genotypes that reduce primaquine metabolism to its active metabolites. Increased primaquine doses have been shown to overcome impaired CYP2D6 function.^50^

Adherence could also be improved by shortening the duration of primaquine from 14 days to 7 days. Low total dose primaquine administered during 7 days is currently recommended in Brazil,^4^ and WHO recently incorporated this regimen as a recommended treatment option.^5^ Two randomised controlled trials have shown the non-inferiority of a 7-day high total dose of primaquine compared with 14-day regimens.^14^, ^32^ Our analysis, which included data from these two trials, supports the efficacy of short-course, high total dose of primaquine. Adherence is related to the duration of treatment;^51^ hence, although efficacy is slightly lower after the 7-day regimen than after the 14-day regimen, the effectiveness of the 7-day regimen might be higher in a real-world setting. A prospective, multicentre, clinical trial is currently studying this aspect (NCT04411836). Comparison of different duration regimens in patients treated with a low total dose of primaquine could not be undertaken due to short follow-up of most patients treated with 7-day regimens.

Administration of higher total doses of primaquine and shorter treatment durations require an increased daily dose of primaquine. Although efficacy is associated with the total primaquine dose, gastrointestinal side-effects, methaemoglobinaemia, and the risk of haemolysis are predominantly associated with the daily primaquine dose.^44^ Our study found that all primaquine doses were associated with increased gastrointestinal symptoms on days 5–7 in children after acute malaria symptoms had resolved; however, only the high daily dose (approximately 1 mg/kg per day) was associated with increased symptoms in patients aged 15 years or older. Coadministration of primaquine with food can reduce gastrointestinal symptoms.^52^ Unfortunately, individual patient data on the administration of food with primaquine were not available for our analysis.

WHO recommends that, where possible, all patients should be tested for G6PD deficiency before commencing primaquine treatment and a daily primaquine regimen should only be administered if the G6PD activity is 30% or higher.^5^ However, high daily primaquine doses might result in clinically relevant haemolysis in individuals with intermediate G6PD deficiency (ie, 30% to <70% enzyme activity), such as heterozygous female patients.^53^ The haematological safety of different primaquine doses and their association with G6PD activity are presented in a separate systematic review and individual patient data meta-analysis.^54^

Our individual patient data meta-analysis was limited by the inclusion of only 40% of eligible studies since 2000; five of the excluded studies were published after Aug 23, 2021, and were not included in the analysis because of the substantial time required to collate individual patient data. However, more than 60% of studies published since 2009 were included, and the sensitivity analyses did not show bias relating to individual study sites. An additional limitation was the need to derive weight-based primaquine mg/kg dosing from the study protocol for almost half of the included patients due to a lack of data on the actual dose administered. Sensitivity analyses restricted to patients with data on the actual dosing showed similar results to the analysis including all patients. The included studies had variable durations of follow-up, leading to the potential for mass censoring of events in the efficacy analyses; however, restriction to studies with a minimum of 180 days of follow-up did not change the results. Methodological limitations of the models used in our analysis include the presence of unmeasured confounding and the inherent potential for bias of HRs.

The presence of genetically related and unrelated relapses, and the lack of standardised methodology to differentiate recurrences into relapses, recrudescences, or reinfections, prevent a direct assessment of the anti-relapse efficacy of primaquine and confound risk estimates from pooling data across different endemic regions. A sensitivity analysis restricted to studies that randomly assigned patients to treatment with or without primaquine was undertaken to ensure reinfection rates across treatment groups were similar; in this a-priori analysis, the results from different treatment groups were similar to those of the primary analysis. This sensitivity analysis also suggested that differences in schizontocidal treatment or primaquine supervision between studies did not lead to confounding.

Our global and subgroup analyses showed a similar reduction in recurrences with a high total dose compared with a low total dose of primaquine; however, the dose–response relationship is likely to be influenced by additional host, parasite, and geographical factors. Therefore, lower doses might be sufficient to achieve cure in some populations. The day-180 endpoint used in our analysis is expected to capture the majority of initial relapses across endemic regions; however, in some regions, such as the Indian subcontinent, a proportion of vivax relapses can occur after 180 days,^55^ and the benefits of increasing the total dose of primaquine might differ. Hence, although patients were included from the Asia-Pacific region, Africa, and the Americas, additional region-focused and country-focused analyses will be needed to inform local antimalarial policy.

In summary, the benefits of a high total dose of primaquine regimens are not restricted to east Asia and Oceania but are apparent in nearly all endemic areas. The reduction of more than 50% of relapses with high-dose primaquine compared with low-dose primaquine could have a profound effect on reducing vivax-associated morbidity and transmission. Shorter course regimens show similar efficacy to 14-day regimens in prospective efficacy studies and thus might improve effectiveness in real-world settings due to improved adherence. However, higher total dose regimens or shortened regimens require an increased daily primaquine dose—higher than the traditional dose of 0·25 mg/kg per day. Doses of 0·5 mg/kg per day were tolerated similarly to 0·25 mg/kg per day, but the benefit of increasing the daily dose to 1 mg/kg needs to be weighed against increases in gastrointestinal symptoms and the potential for haemolysis.^54^

## Data sharing

De-identified participant data used in this analysis are available for access via the WWARN website (https://www.wwarn.org/). Requests for access will be reviewed by a data access committee to ensure that use of data protects the interests of the participants and researchers according to the terms of ethics approval and principles of equitable data sharing. Requests can be submitted by email to malariaDAC@iddo.org via the data access form available at https://www.wwarn.org/working-together/sharing-accessing-data/accessing-data. WWARN is registered with the Registry of Research Data Repositories (https://www.re3data.org/).

## Declaration of interests

JAG and GCKWK are former employees of GSK and hold shares in GSK and AstraZeneca. GCKWK reports travel support from AstraZeneca. JKB reports institutional research funding from Medicines for Malaria Venture, GSK, Wellcome Trust, and Sanaria; participation on the US National Institutes of Health data safety monitoring board; and membership of the editorial board of *Travel Medicine and Infectious Disease* and the guidelines development group for malaria control and elimination, Global Malaria Programme, WHO. RJC, JKB, and RNP report contributions to Up-to-Date. All other authors declare no competing interests.
