# Supplementary material for: Effect of primaquine dose on the risk of recurrence in patients with uncomplicated Plasmodium vivax: a systematic review and individual patient data meta-analysis
Source: Lancet Infect Dis. Author manuscript; Available in PMC 2024 Feb 1. (PMC7615564; doi:10.1016/S1473-3099(23)00430-9)
Supplement: Suppl File [file EMS192937-supplement-Suppl_File.pdf]

THE LANCET
Infectious Diseases

Supplementary appendix

This appendix formed part of the original submission and has been peer reviewed. We post it as supplied by the authors.

Supplement to: Commons RJ, Rajasekhar M, Edler P, et al. Effect of primaquine dose on the risk of recurrence in patients with uncomplicated *Plasmodium vivax*: a systematic review and individual patient data meta-analysis. *Lancet Infect Dis* 2023; published online Sept 22. https://doi.org/10.1016/S1473-3099(23)00430-9.

APPENDIX

Commons RJ, Rajasekhar M *et al*, Effect of primaquine dose on the risk of recurrence in patients with uncomplicated *Plasmodium vivax*: a systematic review and individual patient data meta-analysis

	Page
**Checklist S1.** PRISMA-IPD	3
**Box S1.** Search Strategy	7
**Text S1.** Supplementary methodology	8
**Figure S1.** Directed acyclic graphs for the relationship between primaquine dose (exposure) and outcomes	9
**Table S1.** Studies included in analysis	10
**Table S2.** Study sites included in analysis	11
**Table S3.** Reasons for studies not being included in the efficacy and tolerability analyses	13
**Table S4.** Studies eligible for the efficacy analysis but not included	14
**Figure S2.** Location of study sites for included and eligible but not included studies in efficacy analysis	16
**Figure S3.** Location of study sites by treatment arm in efficacy analysis for the Asia Pacific, Africa and Americas	17
**Figure S4.** Total mg/kg dose of primaquine in patients receiving primaquine	18
**Figure S5.** Total mg/kg dose of primaquine by bodyweight in patients receiving primaquine	19
**Table S5.** Patients planned for 3.5 mg/kg or 7 mg/kg total dose primaquine with subtarget or supratarget dosing	20
**Table S6.** Comparison of baseline characteristics between included and eligible but not included studies in the efficacy analysis	22
**Table S7.** Risk of bias assessment in randomised controlled studies	23
**Table S8.** Risk of bias assessment in single arm observational studies	24
**Figure S6.** Kaplan-Meier figure of cumulative risk of first *P. vivax* recurrence between day 7 and 365 by primaquine treatment category	25
**Table S9.** Risk factors for first *P. vivax* recurrence between days 7 and 180	26
**Table S10.** Sensitivity analyses for relationship between total primaquine mg/kg dose and first *P. vivax* recurrence between days 7 and 180	27
**Table S11.** Sensitivity analysis excluding one study site at a time for relationship between total primaquine mg/kg dose and first *P. vivax* recurrence between days 7 and 180	28
**Figure S7.** Kaplan-Meier cumulative risk of first *P. vivax* recurrence between day 7 and day 42, 90, 180 and 365 by study and primaquine category	29
**Table S12.** Sensitivity analyses for relationship between duration of primaquine and first *P. vivax* recurrence between days 7 and 180 in patients treated with high total doses of primaquine	31
**Table S13.** Sensitivity analyses excluding one study site at a time for relationship between duration of primaquine and first *P. vivax* recurrence between days 7 and 180	32

Table S14. Incidence rate of *P. vivax* recurrences between day 7 and 180 or day 7 and 365 associated with treatment regimen 33
Table S15. Sensitivity analysis for total primaquine dose and incidence rate of all *P. vivax* recurrences between days 7 and 180 34
Table S16. Sensitivity analysis excluding one study site at a time for relationship between total primaquine mg/kg dose and incidence rate of *P. vivax* recurrences between days 7 and 180 35
Table S17. Number of recurrences between days 7 and 180 by treatment arm 36
Table S18. Baseline characteristics of patients included the gastrointestinal tolerability analysis 37
Table S19. Number and percentage of patients reporting gastrointestinal symptoms on days 5-7 39
Figure S8. Association between primaquine daily dose and gastrointestinal symptoms on days 5-7 40
Figure S9. Covariate-adjusted estimated proportion of patients with gastrointestinal symptoms on days 5-7 by treatment arm for all age categories 41
Figure S10. Association between primaquine daily dose and gastrointestinal symptoms on days 5-7 for all age categories 42
Figure S11. Sensitivity analysis of the covariate-adjusted estimated proportion of patients with composite gastrointestinal outcome by treatment arm on days 5-7 43
Figure S12. Sensitivity analysis of the relationship between composite gastrointestinal outcome and primaquine daily dose on days 5-7 44
Table S20. Number and percentage of patients reporting vomiting within an hour of a primaquine dose 45
Figure S13. Covariate-adjusted estimated proportion of patients with gastrointestinal symptoms on days 1-2 by treatment arm 46
Figure S14. Association between primaquine daily dose and gastrointestinal symptoms on days 1-2 47
References S1. Studies not included in analysis 48

Checklist S1. PRISMA-IPD Checklist of items to include when reporting a systematic review and meta-analysis of individual participant data (IPD)

PRISMA-IPD Section/topic	Item No	Checklist item	Reported on page
**Title**			
Title	1	Identify the report as a systematic review and meta-analysis of individual participant data.	1
**Abstract**			
Structured summary	2	Provide a structured summary including as applicable:	7-8
		**Background**: state research question and main objectives, with information on participants, interventions, comparators and outcomes.	
		**Methods**: report eligibility criteria; data sources including dates of last bibliographic search or elicitation, noting that IPD were sought; methods of assessing risk of bias.	
		**Results**: provide number and type of studies and participants identified and number (%) obtained; summary effect estimates for main outcomes (benefits and harms) with confidence intervals and measures of statistical heterogeneity. Describe the direction and size of summary effects in terms meaningful to those who would put findings into practice.	
		**Discussion:** state main strengths and limitations of the evidence, general interpretation of the results and any important implications.	
		**Other:** report primary funding source, registration number and registry name for the systematic review and IPD meta-analysis.	
**Introduction**			
Rationale	3	Describe the rationale for the review in the context of what is already known.	10
Objectives	4	Provide an explicit statement of the questions being addressed with reference, as applicable, to participants, interventions, comparisons, outcomes and study design (PICOS). Include any hypotheses that relate to particular types of participant-level subgroups.	10
**Methods**			
Protocol and registration	5	Indicate if a protocol exists and where it can be accessed. If available, provide registration information including registration number and registry name. Provide publication details, if applicable.	13
Eligibility criteria	6	Specify inclusion and exclusion criteria including those relating to participants, interventions, comparisons, outcomes, study design and characteristics (e.g. years when conducted, required minimum follow-up). Note whether these were applied at the study or individual level i.e. whether eligible participants were included (and ineligible participants excluded) from a study that included a wider population than specified by the review inclusion criteria. The rationale for criteria should be stated.	11
Identifying studies – information sources	7	Describe all methods of identifying published and unpublished studies including, as applicable: which bibliographic databases were searched with dates of coverage; details of any hand searching including of conference proceedings; use of study registers and agency or company databases; contact with the original research team and experts in the field; open adverts and surveys. Give the date of last search or elicitation.	11, appendix p7
Identifying studies – search	8	Present the full electronic search strategy for at least one database, including any limits used, such that it could be repeated.	Appendix p7

Study selection processes	9	State the process for determining which studies were eligible for inclusion.	11
Data collection processes	10	Describe how IPD were requested, collected and managed, including any processes for querying and confirming data with investigators. If IPD were not sought from any eligible study, the reason for this should be stated (for each such study).	11
		If applicable, describe how any studies for which IPD were not available were dealt with. This should include whether, how and what aggregate data were sought or extracted from study reports and publications (such as extracting data independently in duplicate) and any processes for obtaining and confirming these data with investigators.	13, appendix p8, 13-15, 22
Data items	11	Describe how the information and variables to be collected were chosen. List and define all study level and participant level data that were sought, including baseline and follow-up information. If applicable, describe methods of standardising or translating variables within the IPD datasets to ensure common scales or measurements across studies.	12, ref 13
IPD integrity	A1	Describe what aspects of IPD were subject to data checking (such as sequence generation, data consistency and completeness, baseline imbalance) and how this was done.	Ref 13
Risk of bias assessment in individual studies.	12	Describe methods used to assess risk of bias in the individual studies and whether this was applied separately for each outcome. If applicable, describe how findings of IPD checking were used to inform the assessment. Report if and how risk of bias assessment was used in any data synthesis.	Appendix p8
Specification of outcomes and effect measures	13	State all treatment comparisons of interests. State all outcomes addressed and define them in detail. State whether they were pre-specified for the review and, if applicable, whether they were primary/main or secondary/additional outcomes. Give the principal measures of effect (such as risk ratio, hazard ratio, difference in means) used for each outcome.	12-13, appendix p8
Synthesis methods	14	Describe the meta-analysis methods used to synthesise IPD. Specify any statistical methods and models used. Issues should include (but are not restricted to):<br>• Use of a one-stage or two-stage approach.<br>• How effect estimates were generated separately within each study and combined across studies (where applicable).<br>• Specification of one-stage models (where applicable) including how clustering of patients within studies was accounted for.<br>• Use of fixed or random effects models and any other model assumptions, such as proportional hazards.<br>• How (summary) survival curves were generated (where applicable).<br>• Methods for quantifying statistical heterogeneity (such as I^2 and t^2).<br>• How studies providing IPD and not providing IPD were analysed together (where applicable).<br>• How missing data within the IPD were dealt with (where applicable).	13, appendix p8
Exploration of variation in effects	A2	If applicable, describe any methods used to explore variation in effects by study or participant level characteristics (such as estimation of interactions between effect and covariates). State all participant-level characteristics that were analysed as potential effect modifiers, and whether these were pre-specified.	13, appendix p8
Risk of bias across studies	15	Specify any assessment of risk of bias relating to the accumulated body of evidence, including any pertaining to not obtaining IPD for particular studies, outcomes or other variables.	Appendix p8

Additional analyses	16	Describe methods of any additional analyses, including sensitivity analyses. State which of these were pre-specified.	13, appendix p8
**Results**			
Study selection and IPD obtained	17	Give numbers of studies screened, assessed for eligibility, and included in the systematic review with reasons for exclusions at each stage. Indicate the number of studies and participants for which IPD were sought and for which IPD were obtained. For those studies where IPD were not available, give the numbers of studies and participants for which aggregate data were available. Report reasons for non-availability of IPD. Include a flow diagram.	14, 17, Figure 1, appendix pp10-17
Study characteristics	18	For each study, present information on key study and participant characteristics (such as description of interventions, numbers of participants, demographic data, unavailability of outcomes, funding source, and if applicable duration of follow-up). Provide (main) citations for each study. Where applicable, also report similar study characteristics for any studies not providing IPD.	Appendix pp12-12
IPD integrity	A3	Report any important issues identified in checking IPD or state that there were none.	14-15
Risk of bias within studies	19	Present data on risk of bias assessments. If applicable, describe whether data checking led to the up-weighting or down-weighting of these assessments. Consider how any potential bias impacts on the robustness of meta-analysis conclusions.	Appendix pp23-24
Results of individual studies	20	For each comparison and for each main outcome (benefit or harm), for each individual study report the number of eligible participants for which data were obtained and show simple summary data for each intervention group (including, where applicable, the number of events), effect estimates and confidence intervals. These may be tabulated or included on a forest plot.	14-18
Results of syntheses	21	Present summary effects for each meta-analysis undertaken, including confidence intervals and measures of statistical heterogeneity. State whether the analysis was pre-specified, and report the numbers of studies and participants and, where applicable, the number of events on which it is based.	15-18
		When exploring variation in effects due to patient or study characteristics, present summary interaction estimates for each characteristic examined, including confidence intervals and measures of statistical heterogeneity. State whether the analysis was pre-specified. State whether any interaction is consistent across trials.	
		Provide a description of the direction and size of effect in terms meaningful to those who would put findings into practice.	
Risk of bias across studies	22	Present results of any assessment of risk of bias relating to the accumulated body of evidence, including any pertaining to the availability and representativeness of available studies, outcomes or other variables.	Appendix p22
Additional analyses	23	Give results of any additional analyses (e.g. sensitivity analyses). If applicable, this should also include any analyses that incorporate aggregate data for studies that do not have IPD. If applicable, summarise the main meta-analysis results following the inclusion or exclusion of studies for which IPD were not available.	16-18, appendix pp28-29, 31-32, 35, 46-47
**Discussion**			
Summary of evidence	24	Summarise the main findings, including the strength of evidence for each main outcome.	18-22

Strengths and limitations	25	Discuss any important strengths and limitations of the evidence including the benefits of access to IPD and any limitations arising from IPD that were not available.	21-22
Conclusions	26	Provide a general interpretation of the findings in the context of other evidence.	20-22
Implications	A4	Consider relevance to key groups (such as policy makers, service providers and service users). Consider implications for future research.	18-22
**Funding**			
Funding	27	Describe sources of funding and other support (such as supply of IPD), and the role in the systematic review of those providing such support.	23

© Reproduced with permission of the PRISMA IPD Group, which encourages sharing and reuse for non-commercial purposes

Box S1. Search strategy

Search strategy

All prospective *P. vivax* antimalarial clinical trials published between Jan 1, 2000 and June 8, 2023 were identified by the application of the key terms (listed below) through Medline (Pubmed), Web of Science, Embase and the Cochrane Central. Abstracts of all references containing any mention of antimalarial drugs were manually checked to confirm prospective clinical trials, with review of full text when needed. Studies were included if they had active follow up of 28 days or more, included a treatment arm with daily primaquine given over multiple days where primaquine was commenced within 7 days of schizontocidal treatment and was coadministered with chloroquine or one of four artemisinin-based combination therapies (artemether-lumefantrine, artesunate-mefloquine, artesunate-amodiaquine, dihydroartemisinin-piperaquine), or primaquine was given alone. Studies on prevention, prophylaxis, reviews, animal studies, patients with severe malaria, where schizontocidal treatment was unsupervised or where data were extracted retrospectively from medical records outside of a planned trial were excluded. The original review process that this review was based off is documented in more detail in Commons *et al*, Int J Parasitol Drug Drug Res 2017.[12] The year of the study was taken as the year in which the paper was published, although the start and end date of patient enrolment were also recorded. A *post hoc* systematic review for eligible studies in Scopus did not identify any additional eligible studies.

Key terms

Literature search (conducted June 2023) with the following key terms (version undertaken in Pubmed): vivax AND (artefenomel OR arterolane OR amodiaquine OR atovaquone OR artemisinin OR arteether OR artesunate OR artemether OR artemotil OR azithromycin OR artekin OR chloroquine OR chlorproguanil OR cycloguanil OR clindamycin OR coartem OR dapsone OR dihydroartemisinin OR duo-cotecxin OR doxycycline OR halofantrine OR lumefantrine OR lariam OR malarone OR mefloquine OR naphthoquine OR naphthoquinone OR piperaquine OR primaquine OR proguanil OR pyrimethamine OR pyronaridine OR proguanil OR quinidine OR quinine OR riamet OR sulphadoxine OR tetracycline OR tafenoquine).

Text S1. Supplementary methodology

Procedures

Patients treated for 8 or 9 days in the Americas due to greater weights were included in the 7-day primaquine duration group. The primaquine mg base/kg total and daily doses for each individual were calculated from the number of tablets or the base mg doses recorded as administered. If these data were unavailable, doses were calculated from the study protocol or planned dosing regimen.

Food administration with primaquine was categorised according to study protocols as no advice given to patients, patients advised to take primaquine with food and food provided with primaquine. The phenotype of vivax relapses (relapse periodicity) was categorised as low and high by geographic region. A median periodicity of 47 days or lower was defined as high (Battle KE, et al. Geographical variation in Plasmodium vivax relapse. *Malar J* 2014; 13: 144).

Recurrence following *P. vivax* can be caused by relapse due to activation of dormant liver hypnozoites, recrudescence due to failure of schizontocidal therapy to clear blood stage parasites or reinfection with *P. vivax* (Price RN, et al, Phenotypic and genotypic characterisation of drug-resistant *Plasmodium vivax, Trends Parasitol* 2012; 28(11):522; White, NJ *et al.*, The assessment of antimalarial drug efficacy, *Trends Parasitol* 2002; 18(10):458).

Statistical analyses

The minimum study follow up duration was set at to 42 days in the efficacy analysis to reduce bias from including a number of studies that only follow patients for 28 days and thus would contribute a minimum duration of follow up to the outcome. 42 days was chosen to balance inclusion of studies and thus data versus potential risk of bias. Kaplan-Meier survival analyses were left censored (origin) at day 7 and right censored (end time) at the first of: day prior to a >60-day gap between parasite microscopy, day last reviewed or last day of study follow up.

The association between i) target primaquine duration or ii) primaquine dose, and the time to the first vivax recurrence between day 7 and 180 were estimated separately by Cox's proportional hazards regression, with the proportional hazards assumption checked visually. A natural cubic spline model with four knots was used to investigate the relationship between the continuous mg/kg dose of primaquine and the risk of first vivax recurrence between day 7 and 180. Models were adjusted for age, sex and $\log_{10}$ baseline parasite density, with shared frailty for study site, based on a directed acyclic graph (appendix p9). Schizontocidal treatment, relapse periodicity, transmission intensity and primaquine supervision were not included in the efficacy models due to collinearity with study site.

Incidence rates of multiple recurrent episodes of *P. vivax* parasitaemia between day 7 and 180 (and day 365) were calculated from studies with a minimum 180 days follow up that followed patients through multiple episodes of vivax parasitaemia. The incidence rate ratios (IRR) for relapses for primaquine treatment arm were estimated from negative binomial regression models with cluster-robust standard errors, adjusting for age, sex, $\log_{10}$ baseline parasite density and relapse periodicity, with clustering by study site and person-years as offset.

For the tolerability analyses, patient inclusion was restricted to patients treated with primaquine commencing within 3 days of starting schizontocidal treatment. This was done for integrity of the statistical analysis as we assessed the primary composite gastrointestinal outcome on days 5-7. For the secondary composite gastrointestinal outcome on day 1-2, we further restricted inclusion to patients that started primaquine on day 0. To assess whether primaquine exacerbated malaria-associated gastrointestinal symptoms, a generalised estimating equation Poisson model with clustering by study site, exchangeable correlation structure and robust standard error estimates was repeated for the composite outcome on day 1 or 2 in patients treated without primaquine or starting primaquine on day 0. The model was adjusted for age, sex, $\log_{10}$ baseline parasite density, with an interaction term between age and primaquine daily dose.

For all the above regression models, the scales (linear or nonlinear) for the continuous covariates, age and $\log_{10}$ baseline parasite density, were determined based on fractional polynomial regression.

Risk of bias assessment

Within study bias was assessed using the Cochrane Risk of Bias 2 tool (Higgins JPT, Savović J, Page MJ, Elbers RG, Sterne JAC. Assessing risk of bias in a randomized trial. In: Higgins JPT, et al., eds. Cochrane Handbook for Systematic Reviews of Interventions version 63 (updated February 2022): Cochrane; 2022) for randomised controlled trials and the Joanna Briggs Institute Case Series tool (Munn Z, et al., Methodological guidance for systematic reviews of observational epidemiological studies reporting prevalence and cumulative incidence data. *Int J Evid Based Healthc* 2015; **13**(3): 147-53) for single arm studies. Inclusion bias was assessed by comparing baseline characteristics of included studies with studies that were eligible but not included. Heterogeneity of included studies was assessed by removing one study site at a time to calculate the coefficient of variation for the estimated parameter of interest.

A

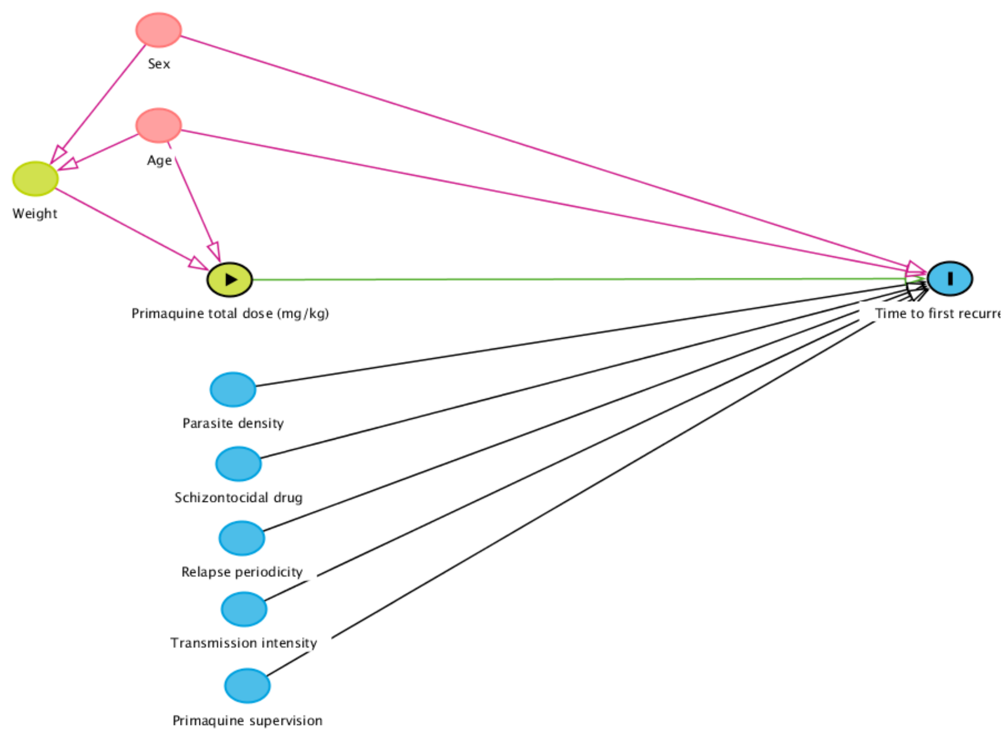

B

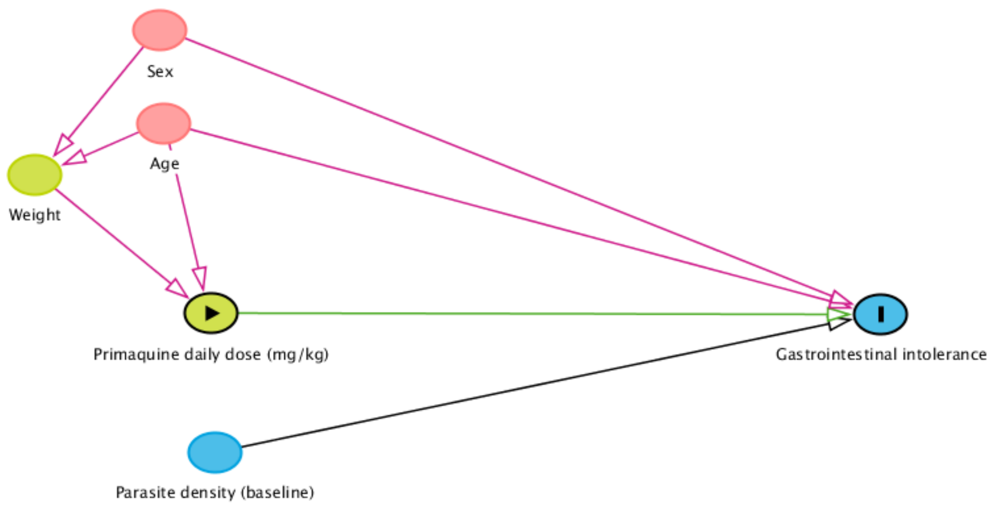

Figure S1. Directed acyclic graphs for the relationship between primaquine dose (exposure) and outcomes. (A) The relationship between total primaquine dose in mg/kg (exposure, green with triangle) and time to first *P. vivax* recurrence (outcome, blue with I) is shown along with the confounders (red) as well as other variables prognostic of the outcome (blue plain). (B) The relationship between daily primaquine dose in mg/kg (exposure, green with triangle) and gastrointestinal intolerance (outcome, blue with I), confounders (red) and other prognostic variables (blue).

Table S1. Studies included in analysis

Author-year	Country	Recruitment Period	Age range	Follow up (days)	Included treatment arms*	PQ duration	PQ taken with food	PQ total dose (mg/kg)	PQ supervision	Randomised	Patients available	Days symptom checklist undertaken and data available	Efficacy	Tolerability
Hasugian-2007[17]	Indonesia	2005	1-56	84	AsAq_Pq_4.2_14d_D2, Dp_Pq_4.2_14d_D2	14 days	No	4.2	No	Yes	150	0, 1, 2, 7, 14	Yes	Yes
Pukrittayakamee-2010[10]	Thailand	1996 – 1998	14-61	28	Pq_3.5_7d_D0, Pq_7.0_7d_D0	7 days	Yes	3.5, 7	Full	Yes	85	0, 1, 2, 3, 4, 5, 6, 7	No	Yes
Barber-2013[18]	Malaysia	2010 – 2015	13-62	42	Cq/ACT +/- Pq	Varied	Recommended	Varied	No	No	39	0	Yes	No
Llanos-Cuentas-2014[19]	Multinational	2010 – 2013	16-72	180	Cq, Cq_Pq_3.5_14d_D1	14 days	Yes	3.5	Partial	Yes	103	0	Yes	No
Pasaribu-2013[20]	Indonesia	2010 – 2012	2-70	365	AsAq_Pq_3.5_14d_D0, Dp_Pq_3.5_14d_D0	14 days	Yes	3.5	Full	Yes	331	0, variable	Yes	Yes
Gonzalez-Ceron-2015[21]	Mexico	2008 – 2010	3-78	365	Cq_Pq_3.5_14d_D0	14 days	Recommended	3.5	Full	No	88	0, 2, 3, 7, 14	Yes	Yes
Lidia-2015[22]	Indonesia	2013	18-88	42	Cq_Pq_3.5_14d_D0, Dp_Pq_3.5_14d_D0	14 days	Yes	3.5	Full	No	51	-	Yes	No
Nelwan-2015[23]	Indonesia	2013	23-49	365	Dp_Pq_7.0_14d_D0	14 days	Yes	7	Full	Yes	56	-	Yes	Yes
Thanh-2015[39]	Vietnam	2009 – 2011	3-60	28	Cq_Pq_5.0_10d_D0	10 days	Yes	5	Full	No	260	0, 1, 2, 3, 7, 14	No	Yes
Yuan-2015[24]	Myanmar	2012 – 2013	1-77	42	Cq_Pq_3.0_8d_D0	8 days	Recommended	3	Partial	No	588	-	Yes	No
Longley-2016[25]	Thailand	2014	7-71	270	Cq_Pq_3.5_14d_D1	14 days	Yes	3.5	Full	No	43	-	Yes	No
Pereira-2016[40]	Brazil	2013 – 2015	19-68	28	Cq_Pq_3.5_7-9d_D0	7-9 days	Recommended	3.5	Partial	No	88	0, 1, 2, 3, 7, 14	No	Yes
Zuluaga-Idarraga-2016[26]	Colombia	2012 – 2013	4-71	180	Cq_Pq_3.5_14d_D0	14 days	Recommended	3.5	Full	No	87	0, 1, 2, 3, 7, 13	Yes	Yes
Abreha-2017[27]	Ethiopia	2012 – 2014	1-67	365	Al, Al_Pq_3.5_14d_D2, Cq, Cq_Pq_3.5_14d_D2	14 days	Yes	3.5	Partial	Yes	397	0, 1, 2, 3, 7, 14	Yes	Yes
Awab-2017[28]	Afghanistan	2009 – 2013	2-84	390	Cq, Cq_Pq_3.5_14d_D0	14 days	Recommended	3.5	Partial	Yes	544	0, 1, 2, 7	Yes	Yes
Chu-2018[29]	Thailand	2010 – 2011	1-63	365	Cq, Cq_Pq_7.0_14d_D0	14 days	No	7	Full	Yes	420	-	Yes	Yes
Daher-2018[30]	Brazil	2012 – 2015	18-65	63	Al_Pq_3.5_7-9d_D0, AsMf_Pq_3.5_7-9d_D0, Cq_Pq_3.5_7-9d_D0	7-9 days	Recommended	3.5	Partial	Yes	264	-	Yes	No
Grigg-2018[31]	Malaysia	2013 – 2015	8m-65	230	Cq/ACT +/- Pq	Varied	No	Varied	No	No	26	-	Yes	No
Chu-2019[32]	Thailand	2012 – 2014	1-63	365	Cq_Pq_7.0_14d_D0, Cq_Pq_7.0_7d_D0, Dp_Pq_7.0_14d_D0, Dp_Pq_7.0_7d_D0	14 days, 7 days, 14 days, 7 days	Yes	7	Full	Yes	654	-	Yes	Yes
de Sena-2019[33]	Brazil	2016 – 2017	2-14	42	Cq_Pq_3.5_7d_D0	7 days	Yes	3.5	Partial	No	113	-	Yes	No
Lacerda-2019[34]	Multinational	2013 – 2017	15-71	180	Cq, Cq_Pq_3.5_14d_D1	14 days	Yes	3.5	Partial	Yes	262	0	Yes	No
Ladeia-Andrade-2019[35]	Brazil	2014 – 2015	7-60	180	Cq_Pq_3.5_7d_D0	7 days	No	3.5	Full	Yes	94	0, 1, 2, 3, 7, 14	Yes	Yes
Llanos-Cuentas-2019[36]	Multinational	2014 – 2017	15-74	180	Cq_Pq_3.5_14d_D1	14 days	Yes	3.5	Partial	Yes	85	0	Yes	Yes
Rijal-2019[37]	Nepal	2015 – 2016	5-75	365	Cq, Cq_Pq_3.5_14d_D0	14 days	Recommended	3.5	Partial	Yes	206	0	Yes	No
Taylor-2019[14]	Multinational	2014 – 2017	9m-94	365	Cq, Cq_Pq_7.0_14d_D0, Cq_Pq_7.0_7d_D0, Dp, Dp_Pq_7.0_14d_D0, Dp_Pq_7.0_7d_D0	14 days, 7 days, 14 days, 7 days	Yes/Recommended (varied by site)	7	Full	Yes	2288	0, 1, 2, 3, 4, 5, 6, 7, 8, 9, 10, 11, 12, 13	Yes	Yes
Karunajeewa-unpublished	Vanuatu	2013	2-35	84	Al, Al_Pq_3.5_14d_D0, Al_Pq_7.0_14d_D0	14 days	Yes	3.5, 7	Full	Yes	26	0, 1	Yes	Yes

ACT – artemisinin-based combination treatment; As – artesunate; Al – artemether-lumefantrine; Aq – amodiaquine; Cq – chloroquine; Dp – dihydroartemisinin-piperaquine; Mf – mefloquine; PQ/Pq – primaquine; *Treatment code describes (schizontocidal drug)_(hypnozoitocidal drug)_(total primaquine dose)_(duration of primaquine treatment eg 14d = 14 days)_(primaquine start day)

Table S2. Study sites included in analysis

Paper	Study site	Country	Region	Lat	Long	Year Start	Year End	MAP Incidence rate (per 1000 persons)	Transmission intensity*	Relapse periodicity†
Hasugian-2007[17]	Timika	Indonesia	Asia-Pacific	-4.61	136.85	2005	2005	22.61	High	High
Pukrittayakamee-2010[10]	Bangkok	Thailand	Asia-Pacific	13.73	100.47	1996	1998	2.15	Moderate‡	High
Barber-2013[18]	Sabah	Malaysia	Asia-Pacific	5.98	116.08	2011	2011	0.21	Low	High
Llanos-Cuentas-2014[19]	Mae Sot	Thailand	Asia-Pacific	16.72	98.58	2011	2013	3.07	Moderate	High
Llanos-Cuentas-2014[19]	Bangkok	Thailand	Asia-Pacific	13.76	100.50	2011	2013	0.16	Low	High
Llanos-Cuentas-2014[19]	Lucknow	India	Asia-Pacific	26.85	80.95	2011	2013	2.84	Moderate	Low
Llanos-Cuentas-2014[19]	Chennai	India	Asia-Pacific	13.06	80.25	2011	2013	1.46	Moderate	Low
Llanos-Cuentas-2014[19]	Manaus	Brazil	Americas	-3.12	-60.02	2011	2013	42.81	High	Low
Llanos-Cuentas-2014[19]	Bikaner	India	Asia-Pacific	28.02	73.31	2011	2013	2.85	Moderate	Low
Llanos-Cuentas-2014[19]	Iquitos	Peru	Americas	-3.74	-73.25	2011	2013	40.49	High	Low
Pasaribu-2013[20]	Tanjung Leidong	Indonesia	Asia-Pacific	2.77	99.98	2011	2011	2.75	Moderate	High
Gonzalez-Ceron-2015[21]	Huehuetan	Mexico	Americas	15.06	-92.33	2008	2009	0.55	Low	Low
Gonzalez-Ceron-2015[21]	Tuxtla Chico	Mexico	Americas	14.91	-92.15	2008	2009	0.55	Low	Low
Gonzalez-Ceron-2015[21]	Fr Hidalgo	Mexico	Americas	14.78	-92.18	2008	2009	0.55	Low	Low
Gonzalez-Ceron-2015[21]	Cacahoatan	Mexico	Americas	15.09	-92.21	2008	2009	0.55	Low	Low
Gonzalez-Ceron-2015[21]	Tapachula	Mexico	Americas	14.91	-92.26	2008	2009	0.55	Low	Low
Lidia-2015[22]	Kupang	Indonesia	Asia-Pacific	-10.18	123.61	2013	2013	15.22	High	High
Nelwan-2015[23]	Sragen	Indonesia	Asia-Pacific	-7.42	111.02	2013	2013	0.07	Low	High
Thanh-2015[39]	Tra Leng	Vietnam	Asia-Pacific	15.28	107.99	2009	2011	0.15	Low	High
Yuan-2015[24]	Laiza	Myanmar	Asia-Pacific	24.75	97.55	2013	2013	9.41	Moderate	High
Longley-2016[25]	Tha Song Yang	Thailand	Asia-Pacific	17.57	97.92	2014	2014	3.09	Moderate	High
Pereira-2016[40]	Rondonia	Brazil	Americas	-11.51	-63.58	2013	2015	11.50	High	Low
Zuluaga-Idarraga-2016[26]	Turbo	Colombia	Americas	8.10	-76.73	2012	2013	5.08	Moderate	Low
Abreha-2017[27]	Batu	Ethiopia	Africa	6.67	39.42	2013	2013	85.49	High	Low
Abreha-2017[27]	Bishoftu	Ethiopia	Africa	8.73	39.01	2013	2013	85.49	High	Low
Awab-2017[28]	Jalalabad	Afghanistan	Asia-Pacific	34.43	70.46	2009	2014	40.12	High	Low
Chu-2018[29]	Mae Sot	Thailand	Asia-Pacific	16.72	98.58	2010	2010	2.15	Moderate	High
Daher-2018[30]	Rondonia	Brazil	Americas	-11.51	-63.58	2013	2014	11.50	High	Low
Daher-2018[30]	Manaus	Brazil	Americas	-3.12	-60.02	2013	2014	30.81	High	Low
Grigg-2018[31]	Kudat	Malaysia	Asia-Pacific	6.89	116.85	2014	2014	0.11	Low	High
Chu-2019[32]	Mae Sot	Thailand	Asia-Pacific	16.72	98.58	2014	2014	3.09	Moderate	High
de Sena-2019[33]	Marajo Island	Brazil	Americas	-0.94	-49.64	2016	2017	5.89	Moderate	Low
Lacerda-2019[34]	Tak	Thailand	Asia-Pacific	16.88	99.13	2013	2016	0.63	Low	High
Lacerda-2019[34]	Rio Tuba	Philippines	Asia-Pacific	8.54	117.44	2013	2016	2.26	Moderate	High
Lacerda-2019[34]	Porto Velho	Brazil	Americas	-8.76	-63.90	2013	2016	9.23	Moderate	Low
Lacerda-2019[34]	Oddar Meanchey Province	Cambodia	Asia-Pacific	14.16	103.82	2013	2016	7.38	Moderate	High
Lacerda-2019[34]	Iquitos	Peru	Americas	-3.74	-73.25	2013	2016	89.80	High	Low
Lacerda-2019[34]	Manaus	Brazil	Americas	-3.12	-60.02	2013	2016	41.14	High	Low
Lacerda-2019[34]	Jimma	Ethiopia	Africa	7.67	36.84	2013	2016	40.53	High	Low
Lacerda-2019[34]	Gondar	Ethiopia	Africa	12.60	37.45	2013	2016	6.72	Moderate	Low
Ladeia-Andrade-2019[35]	Mancio Lima	Brazil	Americas	-7.61	-72.91	2014	2014	47.40	High	Low
Llanos-Cuentas-2019[36]	Umphang	Thailand	Asia-Pacific	15.88	98.92	2015	2016	1.08	Moderate	High
Llanos-Cuentas-2019[36]	Monteria	Colombia	Americas	8.75	-75.88	2015	2016	5.36	Moderate	Low
Llanos-Cuentas-2019[36]	Ho Chi Minh City	Vietnam	Asia-Pacific	10.82	106.63	2015	2016	0.01	Low§	High
Llanos-Cuentas-2019[36]	Thailand4	Thailand	Asia-Pacific			2015	2016	0.13	Low	High
Llanos-Cuentas-2019[36]	Cali	Colombia	Americas	3.45	-76.53	2015	2016	1.93	Moderate	Low
Llanos-Cuentas-2019[36]	Manaus	Brazil	Americas	-3.12	-60.02	2015	2016	18.55	High	Low

Llanos-Cuentas-2019[36]	Iquitos	Peru	Americas	-3.74	-73.25	2015	2016	58.05	High	Low
Rijal-2019[37]	Jhapa	Nepal	Asia-Pacific	26.55	87.89	2016	2016	0.12	Low	High
Rijal-2019[37]	Kailali	Nepal	Asia-Pacific	28.83	80.90	2016	2016	0.22	Low	High
Taylor-2019[14]	Krong Pa	Vietnam	Asia-Pacific	13.22	108.67	2015	2017	0.18	Low	High
Taylor-2019[14]	Dak O	Vietnam	Asia-Pacific	12.00	107.50	2015	2017	0.24	Low	High
Taylor-2019[14]	Bu Gia Map	Vietnam	Asia-Pacific	12.04	107.05	2015	2017	0.24	Low	High
Taylor-2019[14]	Hanura	Indonesia	Asia-Pacific	-5.53	105.24	2015	2017	1.01	Moderate	High
Taylor-2019[14]	Tanjung Leidong	Indonesia	Asia-Pacific	2.77	99.98	2015	2017	1.03	Moderate	High
Taylor-2019[14]	Arba Minch	Ethiopia	Africa	6.01	37.54	2015	2017	17.80	High	Low
Taylor-2019[14]	Laghman	Afghanistan	Asia-Pacific	34.70	70.15	2015	2017	97.70	High	Low
Taylor-2019[14]	Metahara	Ethiopia	Africa	8.90	39.92	2015	2017	26.02	High	Low
Taylor-2019[14]	Jalalabad	Afghanistan	Asia-Pacific	34.43	70.46	2015	2017	116.49	High	Low
Karunajeewa-unpublished	Nambauk	Vanuatu	Asia-Pacific	-15.45	167.08	2013	2013	24.58	High	High
Karunajeewa-unpublished	Port Olry	Vanuatu	Asia-Pacific	-15.04	167.07	2013	2013	24.58	High	High
Karunajeewa-unpublished	Luganville	Vanuatu	Asia-Pacific	-15.51	167.20	2013	2013	24.58	High	High

Lat – latitude; Long – longitude; MAP – malaria Atlas Project; *Transmission intensity is classified as low (an incidence rate of <1 per 1000 persons), moderate (1 to <10 per 1000 persons), high (≥10 per 1000 persons); †Short relapse periodicity ≤47 days; ‡Study done in Bangkok but the majority of patients acquired malaria from the Western border of Thailand where there was high transmission; §Study site in Ho Chi Minh City where there is minimal to no transmission but patients presumed to be from provinces.

Table S3. Reasons for studies not being included in the efficacy and tolerability analyses

Reason	Efficacy analysis		Tolerability analysis	
	Number of studies	Studies*	Number of studies	Studies*
Data not available by August 23, 2021	10	[56-65]	13	[56-64,66-69]
Investigators unable to be contacted	4	[70-73]	8	[70-77]
Missing minimum data for inclusion	2	[78,79]	23	[9,18,19,22,24,25,30,31,33,34,37,78-89]
Initial investigator response but no data provided	4	[90-93]	4	[90-93]
No response from investigators	19	[8,94-111]	37	[8,94-128]
Data available but excluded on patient-level factors	4	[9,80,81,88]	0	

***** References of studies not included are provided in References S1

Table S4. Studies eligible for the efficacy analysis but not included

First Author	Treatment Arms	Number of Sites	Region	Country	Follow up (days)	Randomised	Recruitment period	Treatment arms	Pv patients enrolled	Treated with PQ	Female (%)	Mean Age (SD)	Median Age (range)	Reasons for exclusion
Bergonzoli-2000[70]	4	2	Americas	Costa Rica	180	Yes	1994	Cq_Pq_3.5_14d_D0; Cq_Pq_2.75_9d_D0; Cq_Pq_2.0_5d_D0; Cq_Pq_0.75_1d_D0	132	132	Not stated	30.5 (-)		Unable to be contacted
Abdon-2001[94]	3	1	Americas	Brazil	180	Yes	1994-1995	Cq_Pq_3.5_14d_D0; Cq_Pq_3.5_7d_D0; Cq_Pq_2.5_5d_D0	120	120	37.5	27.3 (-)		No response from investigators
Adak-2001[56]	3	1	Asia-Pacific	India	365	Yes	Not stated	Cq; Cq_Pq_1.25_5d_D3; Cq_Bq	663	220	Not stated	Not stated		Data not available
Dua-2001[95]	1	4	Asia-Pacific	India	540	No	1987-2000	Cq_Pq_1.25_5d_D2	5541	5541	Not stated	Not stated		No response from investigators
Duarte-2001[96]	1	1	Americas	Brazil	180	No	1997-1998	Cq_Pq_3.5_14d_D2	50	50	24	31.8 (12.8)		No response from investigators
Mohapatra-2002[71]	1	1	Asia-Pacific	India	365	No	1998-2000	Cq_Pq_3.5_14d_DX	110	110	36.4	Not stated		Unable to be contacted
Solari Soto-2002[97]	2	1	Americas	Peru	60	Yes	1998-1999	Cq_Pq_3.5_7d_D2; Cq_Pq_3.5_14d_D2	60	60	43.3	26.45 (16.96)		No response from investigators
Yadav-2002[98]	2	1	Asia-Pacific	India	365	Yes	1988-1991	Cq; Cq_Pq_1.25_5d_D2	1482	759	Not stated	Not stated		No response from investigators
Fernandopulle-2003[99]	1	1	Asia-Pacific	Sri Lanka	180	No	Not stated	Cq_Pq_1.25_5d_D0	6	6	Not stated	Not stated		No response from investigators
da Silva-2003[72]	8	1	Americas	Brazil	180	Yes	Not stated	Cq_Pq_3.5_7d_D2; Cq_Pq_2.5_5d_D2; As_Pq_3.5_7d_D2; As_Pq_3.5_7d_D2; As_Pq_3.5_7d_D2; As_Pq_2.5_5d_D2; As_Pq_2.5_5d_D2; As_Pq_2.5_5d_D2	240	240	23.3	32.9 (-)		Unable to be contacted
Rajgor-2003[100]	2	1	Asia-Pacific	India	180	Yes	1998-2000	Cq; Cq_Pq_3.5_14d_D4	273	131	12.1	Not stated		No response from investigators
Walsh-2004[73]	5	1	Asia-Pacific	Thailand	168	Yes	1998-1999	Cq_Tq; Cq_Tq; Cq_Tq; Cq; Cq_Pq_3.5_14d_D2	80	12	68	Not stated		Unable to be contacted
Alvarez-2006[79]	3	2	Americas	Colombia	180	Yes	2001	Cq_Pq_0.75_3d_D1; Cq_Pq_1.75_7d_D1; Cq_Pq_3.5_14d_D1	210	210	33	30.1 (12.8)		Missing minimum data
Carmona-Fonseca-2009[57]	4	2	Americas	Colombia	120	Yes	2001-2003	Cq_Pq_3.5_14d_D1; Cq_Pq_1.75_3d_D1; Cq_Pq_2.5_3d_D1; Cq_Pq_3.5_3d_D1	188	188	30.4	Not stated		Data not available
Orjuela-Sanchez-2009[78]	2	1	Americas	Brazil	336	No	2004-2007	Cq_Pq_3.5_7d_D0; Cq_Pq_3.5_7d_D0	164	164	Not stated	Not stated		Missing minimum data
Carmona-Fonseca-2010[58]	2	1	Americas	Colombia	120	Yes	2005-2008	Cq_Pq_3.5_7d_D1; Cq_Pq_3.5_3d_D1	79	79	Not stated	Not stated		Data not available
Takeuchi-2010[101]	2	1	Asia-Pacific	Thailand	90	Yes	2007-2009	Cq_Pq_3.5_14d_D3; Cq_Pq_3.5_14d_D3	216	216	39.8	Not stated		No response from investigators
Maneeboonyang-2011[102]	2	1	Asia-Pacific	Thailand	90	Yes	2005-2006	Cq_Pq_3.5_14d_D3; Cq_Pq_3.5_14d_D3	92	92	40	Not stated		No response from investigators
Muhamad-2011[103]	1	1	Asia-Pacific	Thailand	42	No	2008-2009	Cq_Pq_3.5_14d_D0	130	130	50.8	Not stated	22	No response from investigators
Van Den Eede-2011[90]	1	1	Americas	Peru	365	No	2008	Cq_Pq_3.5_7d_D0	51	51	49	Not stated	15 (2-80)	Data not provided
Graf-2012[104]	3	1	Americas	Peru	210	Yes	2005-2008	Cq_Pq_2.5_5d_D0; Cq_Pq_3.5_7d_D0; Cq_Pq_3.5_14d_D0	540	540	Not stated	Not stated		No response from investigators

Ganguly-2013[105]	2	1	Asia-Pacific	India	42	Yes	2011-2012	Cq; Cq_Pq_3.5_14d_D0	250	125	10.8	25.2 (-)		No response from investigators
Liu-2013[106]	2	1	Asia-Pacific	China	365	Yes	2009-2010	Cq_Pq_4.0_8d_D0; Anq	260	128	14	Not stated		No response from investigators
Macareo-2013[107]	2	1	Asia-Pacific	Thailand	90	Yes	Not stated	Cq_Pq_7.0_14d_D0; Cq_Tnd	20	6	Not stated	Not stated		No response from investigators
Delgado-Ratto-2014[108]	1	1	Americas	Peru	720	No	2008	Cq_Pq_3.5_7d_D0	37	37	48.6	Not stated	15	No response from investigators
Rajgor-2014[8]		1	Asia-Pacific	India	180	Yes	Not stated	Cq; Cq_Pq_3.5_14d_D4; Cq_Pq_3.5_7d_D4; Cq_Pq_7.0_14d_D4	1556	1159	4.8	31.2 (-)		No response from investigators
Cheoymang-2015[109]	1	1	Asia-Pacific	Thailand	42	No	2008-2009	Cq_Pq_3.5_14d_D1	85	85	34.1	Not stated		No response from investigators
Pareek-2015[110]	3	8	Asia-Pacific	India	180	Yes	Not stated	Cq_Pq_3.5_14d_D3; Cq_Pq_3.5_14d_D3; Cq_Pq_3.5_7d_D3	358	358	17.3	Not stated	20	No response from investigators
Negreiros-2016[91]	1	1	Americas	Brazil	168	No	2014	Cq_Pq_3.5_7d_D0	119	119	45.4	Not stated	23.4 (5-67.3)	Data not provided
Valecha-2016[59]	2	9	Asia-Pacific	India	42	Yes	2011-2013	Cq_Pq_3.5_14d_D3; ArtmPip_Pq_3.5_14d_D3	317	317	8.2	33.7 (13.5)		Data not available
Fukuda-2017[92]	2	1	Asia-Pacific	Thailand	120	Yes	2003-2005	Tq; Cq_Pq_3.5_14d_D3	70	24	17	Not stated	30(20-55)	Data not provided
Mac Donald-Ottevanger-2017[111]	2	2	Americas	Suriname	365	Yes	2006-2008	Cq_Pq_3.5_7d_D3; Cq_Pq_3.5_14d_D3	79	79	34.4	Not stated	24.64 (-)	No response from investigators
Dharmawardena-2017[60]	1	1	Asia-Pacific	Sri Lanka	365	No	2015-2016	Cq_Pq_3.5_14d_D2	32	32	6.8	Not stated	35.5(13-66)	Data not available
Pham-2019[93]	1	4	Asia-Pacific	Vietnam	730	No	2009-2011	Cq_Pq_5.0_10d_D0	260	260	39	Not stated		Data not provided
Poespoprodjo-2022[61]	2	1	Asia-Pacific	Indonesia	180	Yes	2016-2018	Dp_ Pq(sup)_7.0_14d_D0; Dp_ Pq(unsup)_7.0_14d_D0	419	419	47	Not stated	17.2 (7.4-33.1)	Data not available by August 23, 2021
Mekonnen-2023[62]	1	1	Africa	Ethiopia	42	No	2019-2020	Cq_Pq_3.5_14d_D0	102	102	50	Not stated	13.5	Data not available by August 23, 2021
Moore-2023[63]	3	1	Asia-Pacific	PNG	63	Yes	2013-2018	Al_Pq_7.0_14d_D0; Al_Pq_7.0_7d_D0; Al_Pq_7.0_3.5d_D0	73	73	40	Not stated	6.6	Data not available by August 23, 2021
Sutanto-2023[64]	3	2	Asia-Pacific	Indonesia	180	Yes	2018-2019	Dp; Dp_Pq_3.5_14d_D1; Dp_Tq	150	50	0	28.6 (5.6)		Data not available by August 23, 2021
Woon-2023[65]	2	1	Asia-Pacific	PNG	84	Yes	2018-2019	Al_Pq_7.0_3.5d_D3; Al_Pq_7.0_3.5d_D24	219	219	41	Not stated	6.6	Data not available by August 23, 2021

Al – artemether lumefantrine; Anq – artemisinin-naphthoquine; Artm – arterolane maleate; As – artesunate; Bq – bulaquine; Cq – chloroquine; Dp – dihydroartemisinin-piperaquine; Pip – piperaquine; PNG – Papua New Guinea; PQ/Pq – primaquine; Pv – *P. vivax*; SD – standard deviation; sup – supervised; Tnd – tinidazole; Tq – tafenoquine; unsup – unsupervised; *Treatment code describes (schizontocidal drug)_(hypnozoitocidal drug)_(total primaquine dose)_(duration of primaquine treatment eg 14d = 14 days)_(primaquine start day)

Figure S2. Location of study sites for included and eligible but not included studies in efficacy analysis

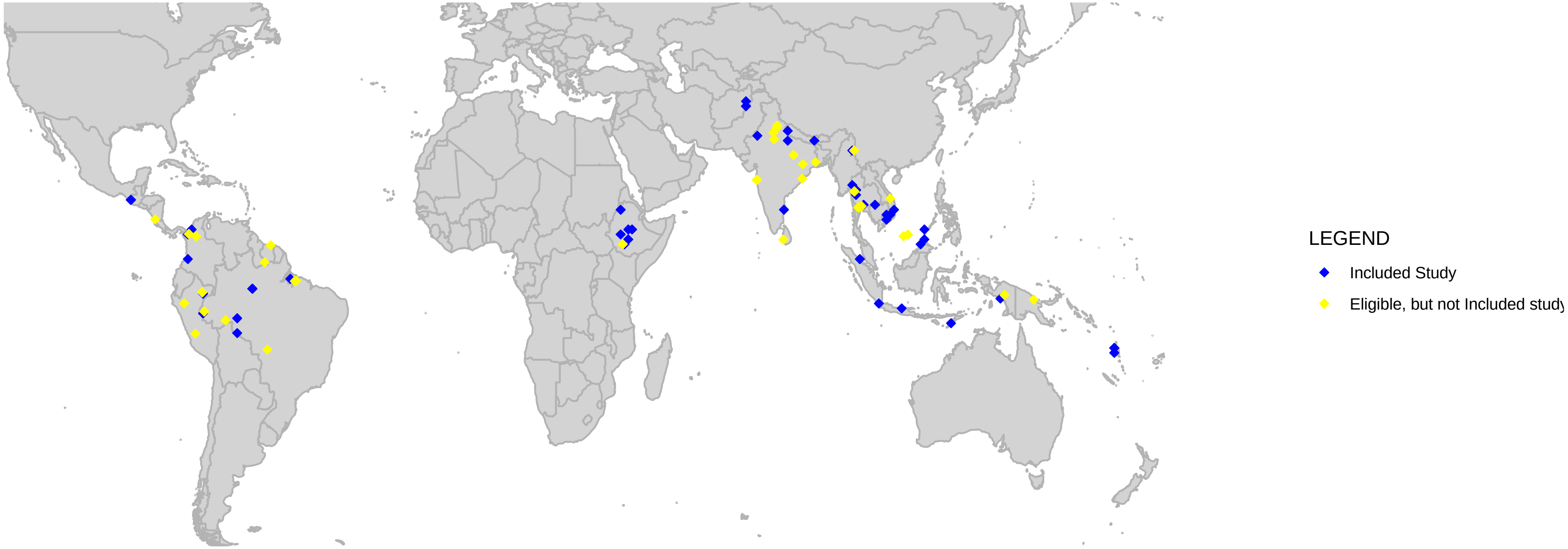

Figure S3. Location of study sites by treatment arm in efficacy analysis for the A) Asia-Pacific, B) Africa and C) Americas

A

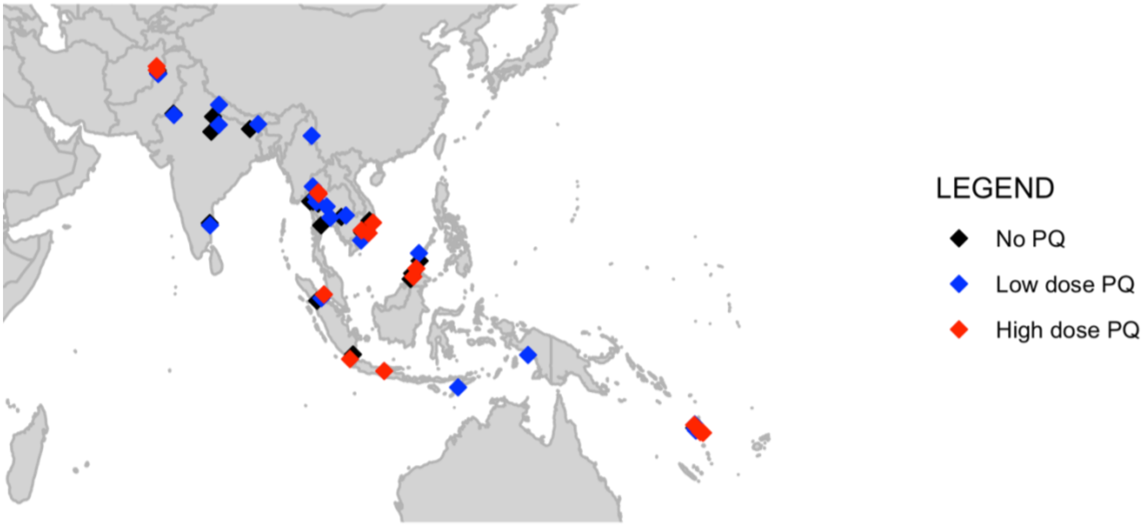

B

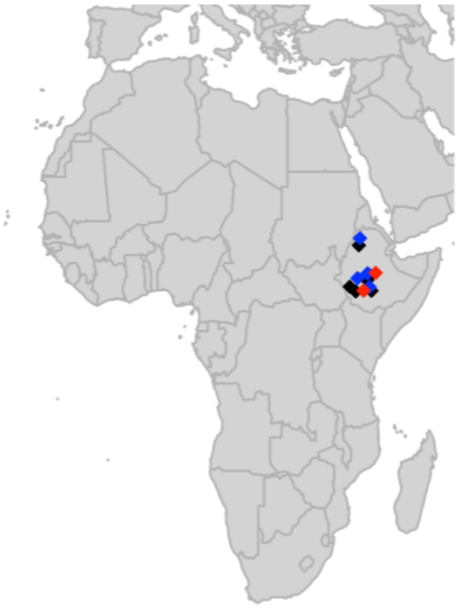

C

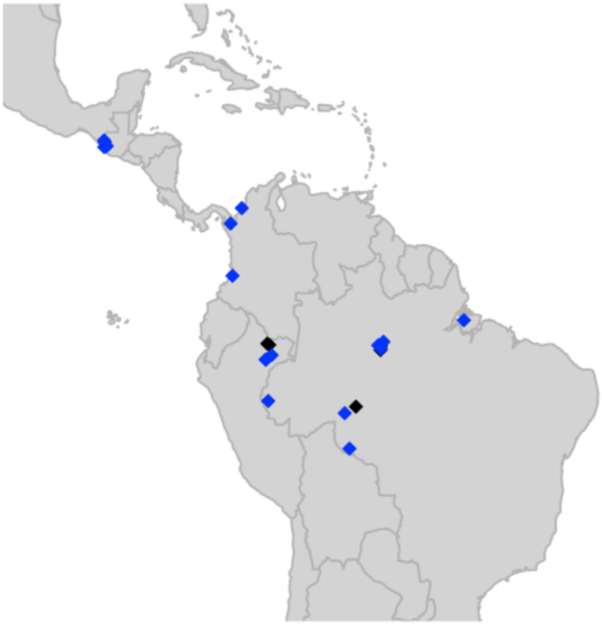

PQ – primaquine; Low dose PQ is a total primaquine dose of 2 to <5 mg/kg; High dose PQ is a total dose of ≥5 mg/kg

Figure S4. Total mg/kg dose of primaquine in patients receiving primaquine

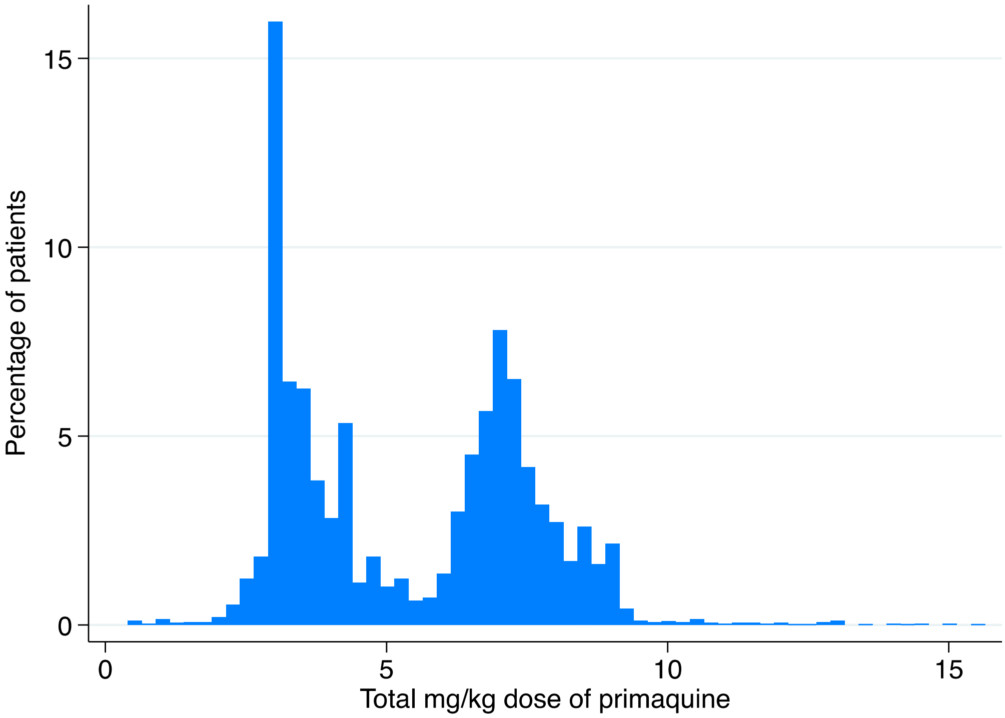

Figure S5. Total mg/kg dose of primaquine by bodyweight in patients receiving primaquine

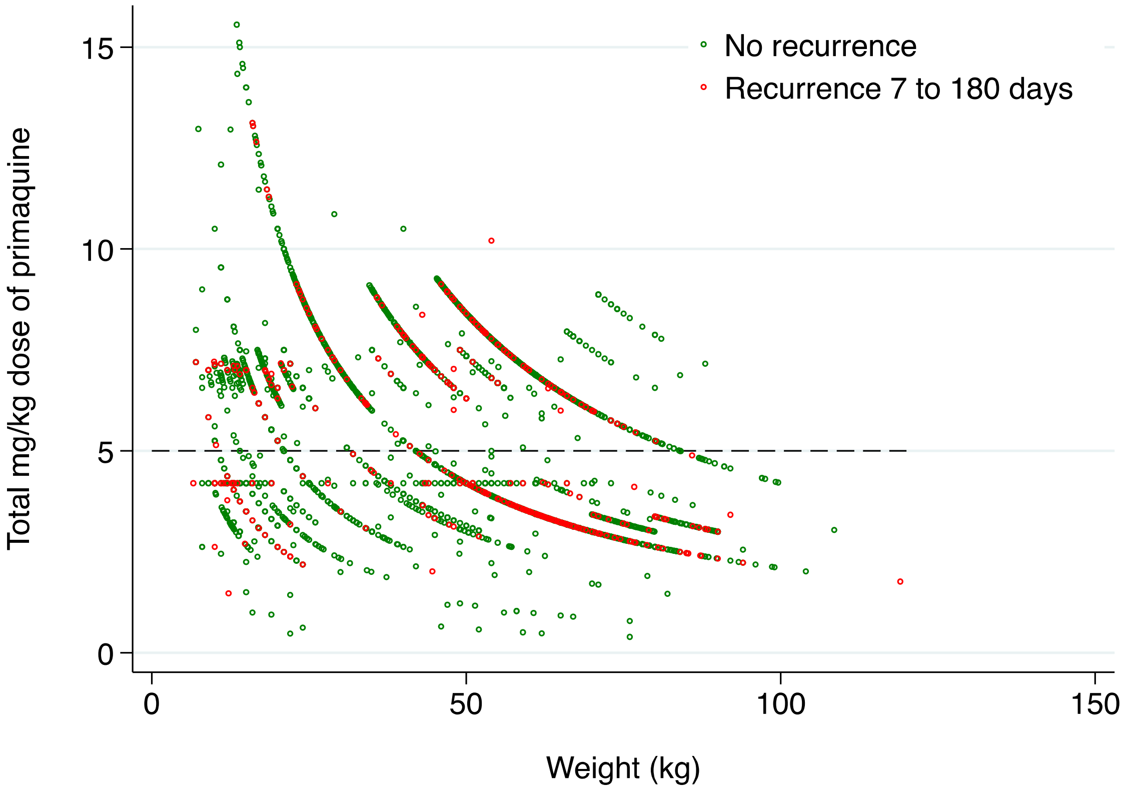

Dashed line: 5 mg/kg dose of primaquine (cut-off between low and high dose primaquine).

Table S5. Patients planned for 3.5 mg/kg or 7 mg/kg total dose primaquine with subtarget or supratarget dosing

	**Expected dosing**<br>N=4,306	**Subtarget dosing**<br>N=128	**Supratarget dosing**<br>N=235
Age (years)			
Median (IQR)	20.0 (12.0-32.8)	22.0 (10.0-37.5)	9.0 (7.0-15.0)
<5	235/4,306 (5.5%)	5/128 (3.9%)	30/235 (12.8%)
5-<15	1,208/4,306 (28.1%)	40/128 (31.2%)	145/235 (61.7%)
≥15	2,863/4,306 (66.5%)	83/128 (64.8%)	60/235 (25.5%)
Sex			
Male	2,727/4,306 (63.3%)	85/128 (66.4%)	125/235 (53.2%)
Female	1,579/4,306 (36.7%)	43/128 (33.6%)	110/235 (46.8%)
Weight (kg)			
Median (IQR)	50.5 (33.0-60.0)	54.2 (29.4-87.3)	22.2 (18.0-40.0)
5 to <15	233/4,259 (5.5%)	3/128 (2.3%)	26/235 (11.1%)
15 to <25	466/4,259 (10.9%)	26/128 (20.3%)	125/235 (53.2%)
25 to <35	412/4,259 (9.7%)	9/128 (7.0%)	11/235 (4.7%)
35 to <45	463/4,259 (10.9%)	7/128 (5.5%)	30/235 (12.8%)
45 to <55	995/4,259 (23.4%)	20/128 (15.6%)	43/235 (18.3%)
55 to <80	1,559/4,259 (36.6%)	18/128 (14.1%)	0/235 (0.0%)
≥80	131/4,259 (3.1%)	45/128 (35.2%)	0/235 (0.0%)
Enrolment variables			
Malnutrition	58/289 (20.1%)	1/7 (14.3%)	9/35 (25.7%)
Primaquine dosing			
Expected dose 3.5 mg/kg	1,778 (41.3%)	45 (35.2%)	94 (40.0%)
Expected dose 7 mg/kg	2,538 (58.7%)	83 (64.8%)	141 (60.0%)
PQ total mg/kg dose, median (IQR)	6.5 (3.6-7.3)	2.4 (2.1-4.2)	9.1 (5.5-9.5)
PQ start day, median (IQR)	0.0 (0.0-0.0)	0.0 (0.0-0.0)	0.0 (0.0-0.0)
Primaquine dose derived from:			
Actual dosing	2,886/4,306 (67.0%)	97/128 (75.8%)	158/235 (67.2%)
Protocol dosing	1,420/4,306 (33.0%)	31/128 (24.2%)	77/235 (32.8%)
Primaquine duration (days)			
7	1,594/4,306 (37.0%)	47/128 (36.7%)	73/235 (31.1%)
14	2,712/4,306 (63.0%)	81/128 (63.3%)	162/235 (68.9%)
PQ supervised			
Unsupervised	0/4,306 (0%)	0/128 (0%)	0/235 (0%)
Partially supervised	1,156/4,306 (26.8%)	40/128 (31.2%)	18/235 (7.7%)
Fully supervised	3,150/4,306 (73.2%)	88/128 (68.8%)	217/235 (92.3%)
Relapse periodicity†			
Low periodicity	1,920/4,306 (44.6%)	84/128 (65.6%)	114/235 (48.5%)
High periodicity	2,386/4,306 (55.4%)	44/128 (34.4%)	121/235 (51.5%)
Geographical region			
Africa	601/4,306 (14.0%)	26/128 (20.3%)	40/235 (17.0%)
Americas	782/4,306 (18.2%)	27/128 (21.1%)	17/235 (7.2%)
Asia-Pacific	2,923/4,306 (67.9%)	75/128 (58.6%)	178/235 (75.7%)

IQR – interquartile range; Data recorded as number (%), median (IQR) or n/N (%); Of 4,669 patients with a target primaquine total dose of 3.5 mg/kg or 7 mg/kg, 128 (2.7%) patients were given a subtarget dose below the expected dosing bounds (2.5-5 mg/kg for 3.5 mg/kg target and 5-9 mg/kg for 7 mg/kg target) and 235 (5.0%) patients were given a supratarget dose above the expected dosing bounds; 47 patients did not have data on weight; 4,021 patients did not have data on malnutrition. *The nutritional status of children aged <5 years of age was calculated as a weight-for-age z-score, using the igrowup package developed by WHO,[38] with z-scores <-2 classified as having malnutrition. Malnutrition status was considered missing if Z scores were <-6 or >6. †Relapse periodicity (the time between vivax relapses) was classified by geographic region as low and high, with high periodicity regions defined as having a median periodicity ≤47 days.[15]

Table S6. Comparison of baseline characteristics between included and eligible but not included studies in the efficacy analysis

Characteristic	Included studies (n=23)	Eligible but not included studies* (n=39)
Region#		
Asia-Pacific, studies (%)	16.3 (71%)	24 (62%)
Africa, studies (%)	1.5 (7%)	1 (3%)
The Americas, studies (%)	5.2 (23%)	14 (36%)
Year of enrolment†		
Pre-2009, studies (%)	1 (4%)	22 (61%)‡
2009-2017, studies (%)	22 (96%)	14 (39%)‡
Follow up duration (days)		
42	4 (17%)	5 (13%)
>42 to <180	3 (13%)	11 (28%)
180	5 (22%)	11 (28%)
>180	11 (48%)	12 (31%)
Age (years), median (IQR)	18 (10-30)	30 (20-31)§
Female, % of patients, median (IQR)	38¶	17 (5-40)||

IQR – interquartile range; # Multinational studies are considered as a proportion of the number of study sites within each region; * Age, and female percentage of targeted studies calculated using frequency weighted mean or median according to number of patients; † Year of enrolment defined as the year study enrolment completed; ‡ Year of enrolment not available for categorisation from 3 studies; § Mean or median age not available from 17 studies; ¶ No IQR presented as data based on actual percentage of female patients in included studies; || Percentage not available from 9 studies.

Table S7. Risk of bias assessment in randomised controlled studies

Author-year	Bias from randomisation	Bias due to deviation from intervention	Bias from missing outcome		Bias in measurement of the outcome		Bias in selection of the reported results	Overall bias	Follow up to 180 days	Comparison of no PQ to PQ
			Efficacy	Tolerability	Efficacy	Tolerability				
Hasugian-2007[17]	Green	Green	Orange	Green	Green	Orange	Orange	Orange	Red	Red
Pukrittayakamee-2010[10]	Green	Green	Green	Green	Green	Orange	Orange	Orange	Red	Red
Pasaribu-2013[20]	Green	Green	Orange	Orange	Green	Orange	Orange	Orange	Green	Red
Llanos-Cuentas-2014[19]	Green	Green	Green	Grey	Green	Grey	Green	Green	Green	Green
Gonzalez-Ceron-2015[21]*	Red	Green †	Orange	Green	Green	Orange	Orange	Red	Green	Red
Lidia-2015[22]*	Red	Green	Green	Grey	Green	Grey	Orange	Red	Red	Red
Nelwan-2015[23]	Green	Green	Orange	Green	Green	Green	Orange	Orange	Green	Red
Abreha-2017[27]	Green	Green	Orange	Green	Green	Orange	Orange	Orange	Green	Green
Awab-2017[28]	Green	Green	Orange	Green	Green	Orange	Orange	Orange	Green	Green
Chu-2018[29]	Green	Green	Orange	Grey	Green	Grey	Orange	Orange	Green	Green
Daher-2018[30]	Green	Green	Orange	Grey	Green	Grey	Green	Orange	Red	Red
Chu-2019[32]	Green	Green †	Orange	Grey	Green	Grey	Orange	Orange	Green	Red
Lacerda-2019[34]	Green	Green	Green	Grey	Green	Grey	Green	Green	Green	Green
Llanos-Cuentas-2019[36]	Green	Green	Green	Green	Green	Green	Green	Green	Green	Green
Ladeia-Andrade-2019[35]	Green	Green	Orange	Green	Green	Orange	Orange	Orange	Green	Red
Rijal-2019[37]	Green	Green †	Orange	Green	Green	Orange	Orange	Orange	Green	Green
Taylor-2019[14]	Green	Green	Green	Green	Green	Green	Green	Green	Green	Green
Karunajeewa-unpublished	Green	Green	Green	Green	Green	Orange	Orange	Orange	Red	Green

Green – low risk of bias; Red – high risk of bias; Orange – unclear risk of bias; Grey – not applicable; Assessed according to the Cochrane Risk of Bias 2 tool for randomised controlled trials (Higgins JPT, Savović J, Page MJ, Elbers RG, Sterne JAC. Assessing risk of bias in a randomized trial. In: Higgins JPT, Thomas J, Chandler J, et al., eds. Cochrane Handbook for Systematic Reviews of Interventions version 63 (updated February 2022): Cochrane; 2022); * Non-randomised quasi-experimental studies; † Studies analysed per protocol but all data available for these meta-analyses; PQ – primaquine.

Table S8. Risk of bias assessment in single arm observational studies

Author-year	Clear criteria for inclusion	Condition measured in reliable way	Valid methods for condition	Consecutive inclusion	Complete inclusion	Demographics reported	Clinical information reported	Outcomes reported	Site description	Analysis appropriate	Follow up to 180 days	Comparison of no PQ to PQ
Barber-2013[18]	Green	Green	Green	Green	Green	Green	Green	Green	Green	Green	Red	Grey
Thanh-2015[39]	Green	Green	Green	Green	Green	Green	Green	Green	Green	Green	Grey	Grey
Yuan-2015[24]	Green	Green	Green	Green	Green	Green	Green	Green	Green	Green	Red	Grey
Longley-2016[25]	Green	Green	Green	Green	Green	Green	Green	Green	Green	Green	Green	Grey
Pereira-2016[40]	Green	Green	Green	Green	Green	Green	Green	Green	Green	Green	Red	Grey
Zuluaga-Idarraga-2016[26]	Green	Green	Green	Green	Green	Green	Green	Green	Green	Green	Green	Grey
Grigg-2018[31]	Orange	Green	Green	Orange	Orange	Red	Red	Green	Green	Green	Red	Grey
de Sena-2019[33]	Green	Green	Green	Green	Green	Green	Green	Green	Green	Green	Red	Grey

Green – yes (low risk of bias); Red – no (higher risk of bias); Orange – unclear; Grey – not applicable; Assessed according to the Joanna Briggs Institute Case Series tool (Munn Z, Moola S, Lisy K, Riitano D, Tufanaru C. Methodological guidance for systematic reviews of observational epidemiological studies reporting prevalence and cumulative incidence data. *Int J Evid Based Healthc* 2015; 13(3): 147-53) for single arm studies; The appropriateness of analysis was considered appropriate for all studies given that the individual patient data were re-analysed as part of these meta-analyses; PQ – primaquine.

Figure S6. Kaplan-Meier figure of cumulative risk of first *P. vivax* recurrence between day 7 and 365 by treatment regimen

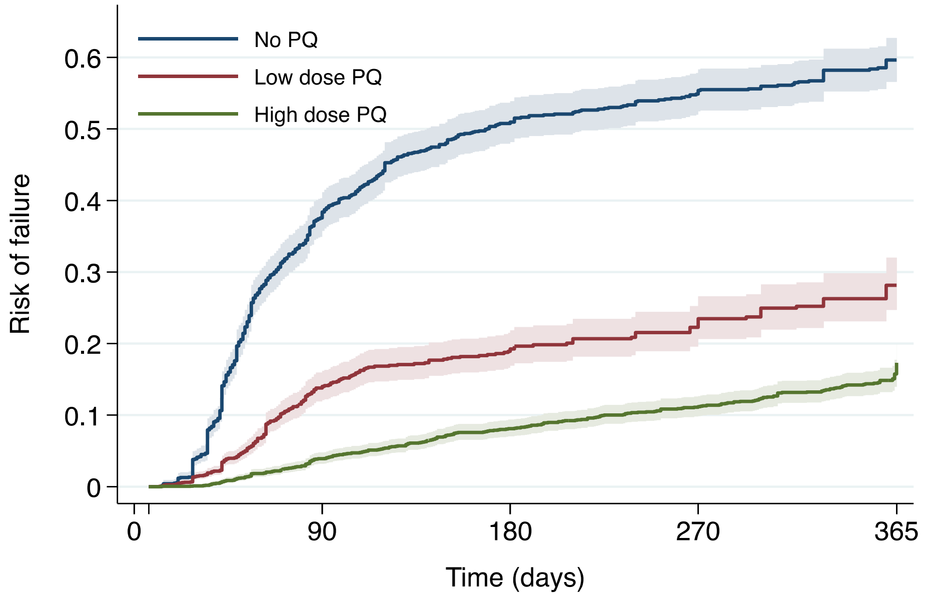

Number at risk

	0	90	180	270	365
No PQ	1423	733	460	375	159
Low dose PQ	2355	749	476	319	184
High dose PQ	2780	2105	1610	1340	216

Shaded area – 95% confidence intervals; PQ – primaquine; Low dose PQ is a total primaquine dose of 2 to <5 mg/kg; High dose PQ is a total dose of ≥5 mg/kg. Log rank test comparing no PQ to low dose PQ p=<0.0001; log rank test comparing no PQ to high dose PQ p=<0.0001; log rank test comparing low dose PQ to high dose PQ p=<0.0001. By day 180, in the no PQ arm there were 628 (44.1%) patients with recurrences and 335 (23.5%) patients censored, in the low dose PQ arm there were 234 (9.9%) patients with recurrences and 1,645 (69.9%) patients censored and in the high dose PQ arm there were 183 (6.6%) patients with recurrences and 987 (35.5%) patients censored.

Table S9. Risk factors for first *P. vivax* recurrence between days 7 and 180

	Total N (n)	Crude hazard ratio (95% CI)	p-value	Adjusted hazard ratio* (95% CI)	p-value
Total primaquine dose					
None	628/1,470	Reference	-	Reference	-
Low	183/2,569	0.21 (0.16, 0.26)	<0.0001	0.21 (0.17, 0.27)	<0.0001
High	234/2,811	0.10 (0.08, 0.12)	<0.0001	0.10 (0.08, 0.12)	<0.0001
Primaquine duration					
7-9 days	168/2,289	Reference	-		
14 days	247/3,059	0.75 (0.56, 1.02)	0.07		
Primaquine supervision					
Fully supervised	233/3,433	Reference	-		
Unsupervised	30/147	15.17 (2.89, 79.50)	0.001		
Partially supervised	154/1,800	1.10 (0.51, 2.38)	0.80		
Age, every 5 year increase	1,045/6,850	0.95 (0.93, 0.97)	<0.0001	0.94 (0.92, 0.97)	<0.0001
Age category, years					
≥15	673/4,193	Reference	-		
<5	93/466	1.52 (1.20, 1.91)	0.0004		
5 to <15	279/2,191	1.03 (0.88, 1.21)	0.69		
Weight, every 5 kg increase	1,045/6,850	0.98 (0.96, 1.00)	0.05		
Sex					
Male	670/4,236	Reference	-	Reference	-
Female	375/2,614	0.93 (0.82, 1.06)	0.30	0.96 (0.84, 1.09)	0.51
Haemoglobin, every 1 g/dL increase	991/6,025	0.97 (0.94, 1.01)	0.17		
Parasitaemia, parasites/μL every 10-fold increase	1,045/6,850	1.42 (1.28, 1.58)	<0.0001	1.33 (1.20, 1.48)	<0.0001
Presence of fever	1,000/6,280	1.06 (0.80, 1.41)	0.67		
Rapid schizontocidal elimination half-life	1,045/6,850	1.40 (1.06, 1.85)	0.02		
Chloroquine dose, every 5 mg/kg increase	764/4,594	1.01 (0.91, 1.11)	0.86		
Relapse periodicity					
Low	547/2,909	Reference	-		
High	498/3,941	1.27 (0.67, 2.38)	0.46		
Region					
Asia-Pacific	587/4,920	Reference	-		
Africa	243/987	0.99 (0.38, 2.62)	0.99		
Americas	215/943	1.18 (0.59, 2.35)	0.65		
Transmission intensity					
Low	93/811	Reference	-		
Moderate	385/3,246	0.50 (0.22, 1.15)	0.10		
High	567/2,793	1.69 (0.80, 3.57)	0.17		

CI – Confidence Interval; Crude and adjusted hazard ratios include clustering for study site. *Multivariable analysis: Weight was excluded due to collinearity with age; Relapse periodicity, geographical region and transmission intensity were not included due to their collinearity with study site and the expectation that they would not satisfy the proportional hazards assumption due to differing time to first relapse in differing regions; Baseline haemoglobin and temperature were not included due to less complete data and not considered *a priori* to be a confounder of the association between total primaquine dose and *P. vivax* recurrence.

Table S10. Sensitivity analyses for relationship between total primaquine mg/kg dose and first *P. vivax* recurrence between days 7 and 180

	Baseline results				Adjusted Hazards Ratio (AHR, 95% CI)			
	Recurrence between day 7 and 180	Recurrence between day 28 and 180*	Patients where actual PQ dose recorded	Patients where PQ dose was fully supervised	Patients treated with chloroquine	Studies with ≥180 days follow up	Studies which randomised treatment to PQ versus no PQ	Analysis without shared frailty
	n=6,850	n=5,836	n=4,617	n=4,116	n=4,541	n=5,657	n=4,139	n=6,850
Total primaquine dose								
None	Reference	Reference	Reference	Reference	Reference	Reference	Reference	Reference
Low	0.21 (0.17, 0.27)	0.22 (0.17, 0.28)	0.31 (0.18, 0.54)	0.24 (0.13, 0.44)	0.21 (0.16, 0.27)	0.20 (0.16, 0.26)	0.21 (0.16, 0.27)	0.31 (0.26, 0.36)
High	0.10 (0.08, 0.12)	0.10 (0.08, 0.12)	0.10 (0.08, 0.12)	0.10 (0.08, 0.12)	0.08 (0.06, 0.10)	0.09 (0.08, 0.12)	0.10 (0.08, 0.12)	0.11 (0.09, 0.13)

CI – Confidence Interval; PQ – primaquine; *A priori* sensitivity analyses for multivariable Cox regression model adjusted for age, sex and (log) baseline parasite density with shared frailty for study site. * The efficacy outcome was restricted to recurrences between day 28 and 180 (assuming 28 days of post-treatment prophylaxis for all treatments).

Table S11. Sensitivity analysis excluding one study site at a time for relationship between total primaquine mg/kg dose and first *P. vivax* recurrence between days 7 and 180

Variable	Range of AHR	Coefficient of Variation (%)
Total primaquine dose		
None	Reference	-
Low	0.19-0.23	2.56
High	0.08-0.10	2.40

AHR – Adjusted Hazard Ratio; Sensitivity analysis for multivariable Cox regression model adjusted for age, sex and (log) baseline parasite density with shared frailty for study site.

Figure S7. Kaplan-Meier cumulative risk of first *P. vivax* recurrence between day 7 and A) day 42, B) day 90, C) day 180 and D) day 365 by study and primaquine category

A

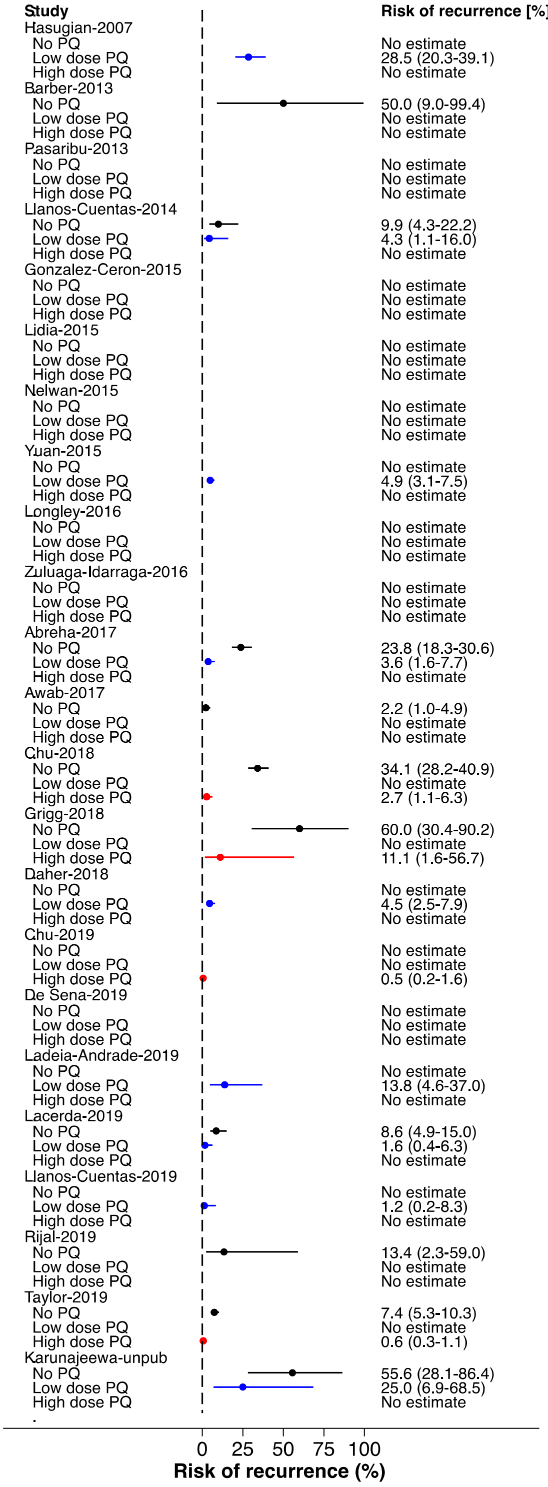

B

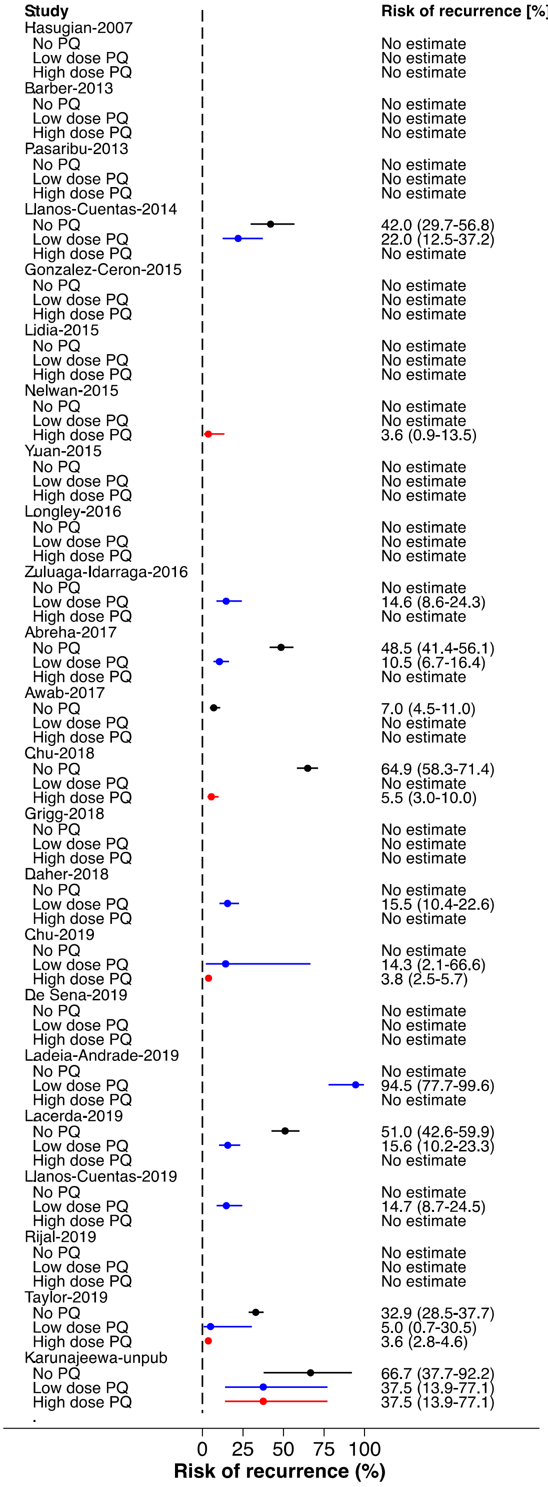

C

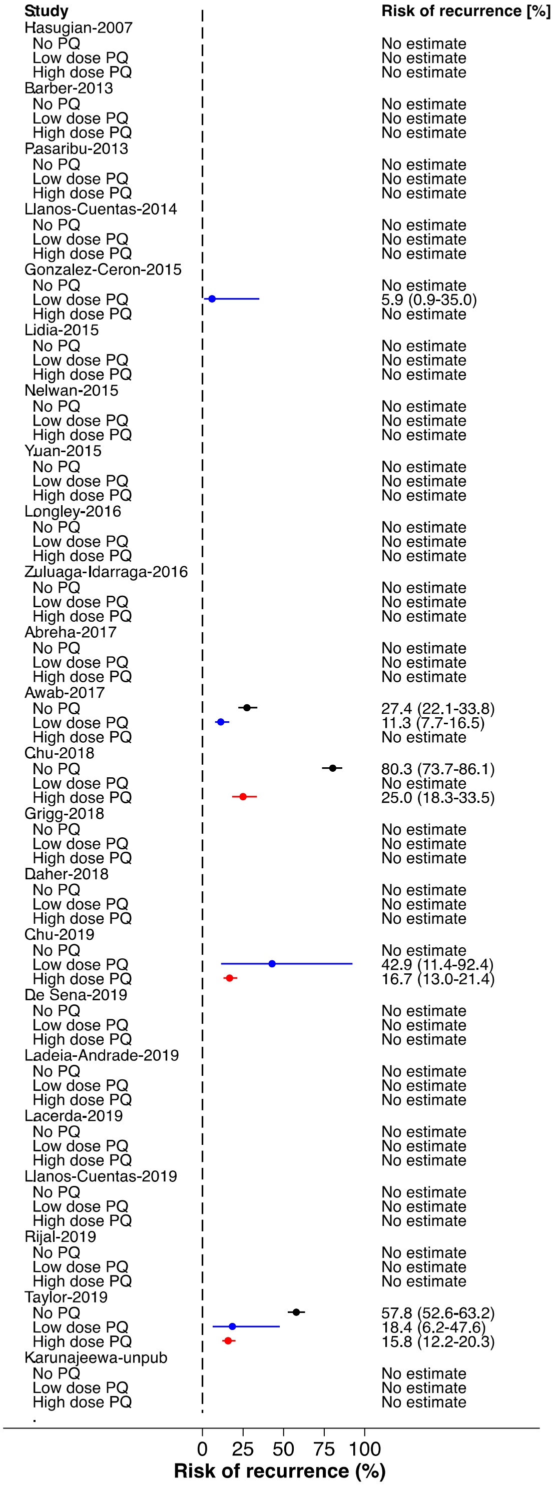

D

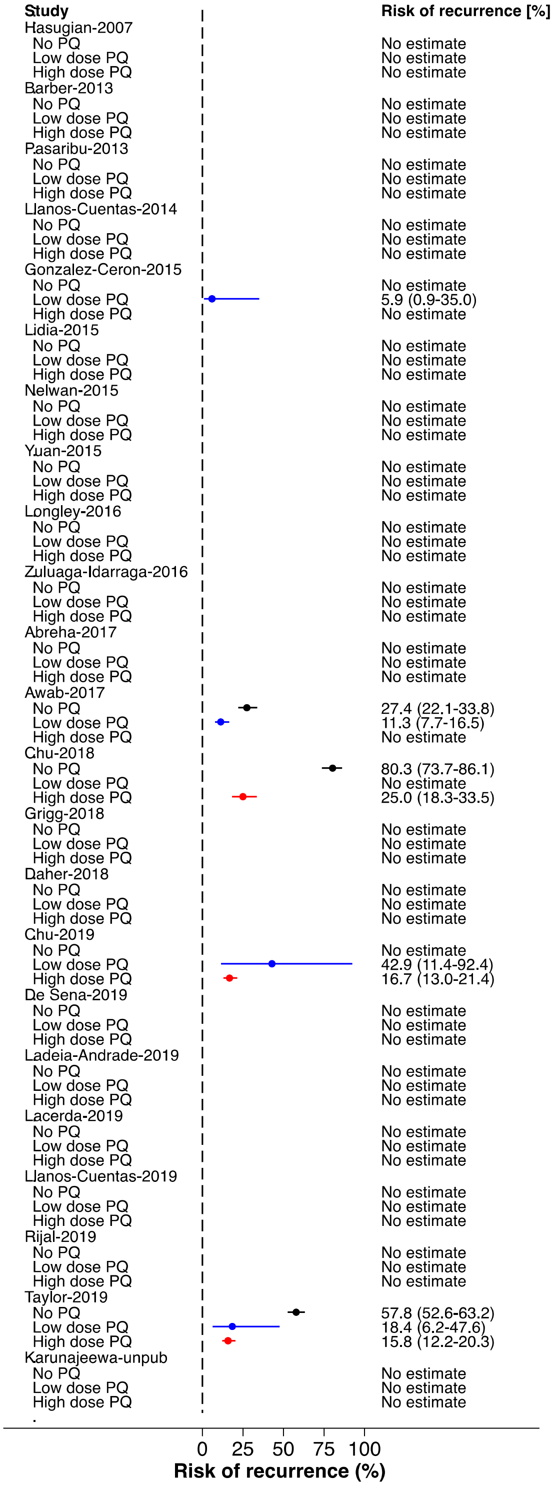

PQ – primaquine; Low dose PQ is a total primaquine dose of 2 to <5 mg/kg; High dose PQ is a total dose of ≥5 mg/kg; Day 90 cumulative risk for Karunajeewa-unpublished was calculated at day 84.

Table S12. Sensitivity analyses for relationship between duration of primaquine and first *P. vivax* recurrence between days 7 and 180 in patients treated with high total dose primaquine

	Baseline results	Adjusted hazard ratio (AHR, 95% CI)					
	**Recurrence between day 7 and 180**	**Recurrence between day 28 and 180***	**Patients where actual PQ dose recorded**	**Patients treated with chloroquine**	**Studies with ≥180 days follow up**	**Studies which compared PQ versus no PQ**	**Analysis without shared frailty**
	**n=2,811**	**n=2,600**	**n=2,716**	**n=1,585**	**n=2,768**	**n=1,971**	**n=2,811**
Primaquine duration							
7 days	Reference	Reference	Reference	Reference	Reference	Reference	Reference
14 days	0.80 (0.59-1.09)	0.81 (0.59, 1.10)	0.80 (0.58, 1.09)	0.91 (0.60, 1.36)	0.78 (0.57, 1.06)	0.76 (0.53, 1.09)	0.88 (0.66, 1.18)

CI – Confidence Interval; PQ – primaquine; *A priori* sensitivity analyses for multivariable Cox regression model adjusted for primaquine mg/kg total dose, age, sex and (log) baseline parasite density with shared frailty for study site. * The efficacy outcome was restricted to recurrences between day 28 and 180 (assuming 28 days of post-treatment prophylaxis for all treatments).

Table S13. Sensitivity analyses excluding one study site at a time for relationship between duration of primaquine and first *P. vivax* recurrence between days 7 and 180 180 in patients treated with high total dose primaquine

Variable	Range of AHR	Coefficient of Variation (%)
Primaquine duration		
7 days	Reference	-
14 days	0.76-0.84	1.93

AHR – adjusted hazard ratio; Sensitivity analysis for multivariable Cox regression model adjusted for primaquine mg/kg total dose, age, sex and (log) baseline parasite density with shared frailty for study site.

Table S14. Incidence rate of *P. vivax* recurrences between day 7 and 180 or day 7 and 365 associated with treatment regimen

Primaquine dose	Recurrences / person years	Incidence rate (95%CI, recurrences per person year)	Unadjusted*		Adjusted†	
			IRR (95%CI)	p value	IRR (95%CI)	p value
All recurrences by day 180						
No PQ	1,101/599·0	1·84 (1·74-1·95)	Reference		Reference	
Low dose PQ	205/437·2	0·47 (0·42-0·52)	0·26 (0·14-0·47)	<0·0001	0·27 (0·17-0·43)	<0·0001
High dose PQ	229/1151·5	0·20 (0·18-0·22)	0·11 (0·06-0·20)	<0·0001	0·11 (0·06-0·21)	<0·0001
All recurrences by day 365						
No PQ	1,525/983·3	1·55 (1·50-1·61)	Reference		Reference	
Low dose PQ	268/615·5	0·44 (0·40-0·48)	0·29 (0·16-0·50)	<0·0001	0·31 (0·21-0·46)	<0·0001
High dose PQ	406/1979·6	0·21 (0·19-0·22)	0·13 (0·07-0·22)	<0·0001	0·13 (0·07-0·23)	<0·0001
Symptomatic recurrences by day 180						
No PQ	535/644·4	0·83 (0·80-0·86)	Reference		Reference	
Low dose PQ	110/486·9	0·23 (0·19-0·27)	0·27 (0·17-0·43)	<0·0001	0·26 (0·18-0·39)	<0·0001
High dose PQ	173/1206·3	0·14 (0·12-0·16)	0·17 (0·11-0·27)	<0·0001	0·20 (0·14-0·29)	<0·0001
Symptomatic recurrences by day 365						
No PQ	713/1102·9	0·65 (0·62-0·67)	Reference		Reference	
Low dose PQ	157/718·6	0·22 (0·19-0·25)	0·33 (0·23-0·49)	<0·0001	0·32 (0·23-0·45)	<0·0001
High dose PQ	301/2176·8	0·14 (0·12-0·15)	0·21 (0·14-0·32)	<0·0001	0·24 (0·17-0·35)	<0·0001

IRR – Incidence Rate Ratio; PQ – primaquine; Low dose PQ is a total primaquine dose of 2 to <5 mg/kg; High dose PQ is a total dose of ≥5 mg/kg; Person-years of observation (PYO) started at day 7 and stopped at the last visit before the outcome day or the outcome day if they had additional visits after this. The incidence rate of symptomatic recurrences was calculated using the entire period between start and stop dates to determine the PYO, while the PYO for the incidence rate of any recurrence (symptomatic or asymptomatic) excluded days where a gap between blood smears was >30 days. *With clustering for study site; †Adjusted for age, sex, (log) baseline parasite density and relapse periodicity with clustering for study site.

Table S15. Sensitivity analyses for total primaquine dose and incidence rate of all *P. vivax* recurrences between days 7 and 180

Variable	Baseline model	IRR (95% CI)	
	All recurrences between days 7 and 180 (adjusted) n=5,448	Patients where actual PQ dose recorded n=4,278	Analysis without clustering by study site n=5,448
Total primaquine dose			
None	Reference	Reference	Reference
Low	0.27 (0.17, 0.43)	0.65 (0.49, 0.86)	0.27 (0.23, 0.32)
High	0.11 (0.06, 0.21)	0.10 (0.09, 0.12)	0.11 (0.09, 0.12)

CI – Confidence Interval; IRR – Incidence Rate Ratio; PQ - primaquine; Negative binomial regression model includes confounders age, sex, (log) baseline parasite density and relapse periodicity, and clustering for study site unless stated otherwise.

Table S16. Sensitivity analysis excluding one study site at a time for relationship between total primaquine mg/kg dose and incidence rate of *P. vivax* recurrences between days 7 and 180

Variable	Range of IRR	Coefficient of Variation (%)
Total primaquine dose		
None	Reference	-
Low	0.24-0.33	4.87
High	0.09-0.17	9.70

IRR – Incidence Rate Ratio; Negative binomial regression model includes confounders age, sex, (log) baseline parasite density and relapse periodicity, and clustering for study site.

Table S17. Number of recurrences between days 7 and 180 by treatment arm

Number of recurrences	No primaquine N=1,433 n (%)	Low dose primaquine N=1,323 n (%)	High dose primaquine N=2,692 n (%)
0	796 (55.5%)	1,162 (87.8%)	2,480 (92.1%)
1	356 (24.8%)	125 (9.4%)	196 (7.3%)
≥2	281 (19.6%)	36 (2.7%)	16 (0.6%)

Low dose PQ is a total primaquine dose of 2 to <5 mg/kg; High dose PQ is a total dose of ≥5 mg/kg;

Table S18. Baseline characteristics of patients included in the gastrointestinal tolerability analysis

	Overall	No primaquine	Low daily dose primaquine (<0.375 mg/kg/day)	Intermediate daily dose primaquine (≥0.375 & <0.75 mg/kg/day)	High daily dose primaquine (≥0.75 mg/kg/day)
	N=5,609	N=1,156	N=1,127	N=1,777	N=1,549
Sex					
Male	3,578 (63.8%)	754 (65.2%)	683 (60.6%)	1,175 (66.1%)	966 (62.4%)
Female	2,031 (36.2%)	402 (34.8%)	444 (39.4%)	602 (33.9%)	583 (37.6%)
Age (years)					
Median (IQR)	17.6 (10.2-29.0)	17.0 (10.0-27.0)	19.0 (11.0-34.0)	18.4 (11.0-30.0)	16.0 (10.0-25.0)
<5	406 (7.2%)	86 (7.4%)	96 (8.5%)	101 (5.7%)	123 (7.9%)
5-<15	1,823 (32.5%)	389 (33.7%)	312 (27.7%)	546 (30.7%)	576 (37.2%)
≥5	3,380 (60.3%)	681 (58.9%)	719 (63.8%)	1,130 (63.6%)	850 (54.9%)
Enrolment variables					
Weight (kg)	47.0 (26.2-57.3)	47.0 (26.9-57.0)	51.0 (30.0-63.0)	48.0 (28.7-57.8)	43.0 (23.0-54.0)
Malnutrition*	125/500 (25.0%)	25/101 (24.8%)	23/119 (19.3%)	32/129 (24.8%)	45/151 (29.8%)
Presence of fever	5,147/5,526 (93.1%)	1,076/1,153 (93.3%)	1,053/1,111 (94.8%)	1,587/1,714 (92.6%)	1,431/1,548 (92.4%)
Parasitaemia, parasites/mL (median (IQR))	3584.0 (1160.0-9640.0)	3878.9 (1600.0-10160.0)	2800.0 (840.0-6360.0)	3632.0 (1062.0-10225.9)	4080.0 (1185.2-11540.7)
Haemoglobin, g/dL†	12.6 (1.9)	12.7 (1.8)	12.3 (2.0)	12.7 (1.7)	12.6 (1.9)
Schizontocidal treatment					
Chloroquine	3,507 (62.5%)	845 (73.1%)	631 (56.0%)	1,072 (60.3%)	959 (61.9%)
Artemether-lumefantrine	226 (4.0%)	115 (9.9%)	94 (8.3%)	10 (0.6%)	7 (0.5%)
Artesunate-amodiaquine	229 (4.1%)	0 (0.0%)	203 (18.0%)	26 (1.5%)	0 (0.0%)
Dihydroartemisinin-piperaquine	1,562 (27.8%)	196 (17.0%)	199 (17.7%)	626 (35.2%)	541 (34.9%)
None	85 (1.5%)	0 (0.0%)	0 (0.0%)	43 (2.4%)	42 (2.7%)
Primaquine dosing					
PQ total mg/kg dose	6.6 (4.2-7.3)		3.8 (3.2-4.2)	7.0 (6.2-7.6)	7.0 (6.3-7.7)
PQ start day					
0	4,066 (91.3%)		746 (66.2%)	1,771 (99.7%)	1,549 (100.0%)
1	85 (1.9%)		84 (7.5%)	1 (0.1%)	0 (0.0%)
2	302 (6.8%)		297 (26.4%)	5 (0.3%)	0 (0.0%)
Primaquine duration (days)					
7-10	1,768 (39.7%)		20 (1.8%)	227 (12.8%)	1,521 (98.2%)
14	2,685 (60.3%)		1,107 (98.2%)	1,550 (87.2%)	28 (1.8%)
Primaquine dose derived from					
Actual dosing	3,288 (73.8%)		201 (17.8%)	1,583 (89.1%)	1,504 (97.1%)
Protocol dosing	1,165 (26.2%)		926 (82.2%)	194 (10.9%)	45 (2.9%)
PQ supervised					
Unsupervised	115 (2.6%)		115 (10.2%)	0 (0.0%)	0 (0.0%)
Partially supervised	632 (14.2%)		533 (47.3%)	96 (5.4%)	3 (0.2%)
Fully supervised	3,706 (83.2%)		479 (42.5%)	1,681 (94.6%)	1,546 (99.8%)
PQ administered with food					

No	407 (9.1%)		121 (10.7%)	281 (15.8%)	5 (0.3%)
Yes	2,791 (62.7%)		572 (50.8%)	1,034 (58.2%)	1,185 (76.5%)
Recommended	1,255 (28.2%)		434 (38.5%)	462 (26.0%)	359 (23.2%)
Relapse periodicity‡					
Low periodicity	2,348 (41.9%)	665 (57.5%)	683 (60.6%)	585 (32.9%)	415 (26.8%)
High periodicity	3,261 (58.1%)	491 (42.5%)	444 (39.4%)	1,192 (67.1%)	1,134 (73.2%)
Transmission intensity§					
Low	830 (14.8%)	64 (5.5%)	103 (9.1%)	236 (13.3%)	427 (27.6%)
Moderate	2,471 (44.1%)	418 (36.2%)	385 (34.2%)	961 (54.1%)	707 (45.6%)
High	2,308 (41.1%)	674 (58.3%)	639 (56.7%)	580 (32.6%)	415 (26.8%)
Region					
Africa	961 (17.1%)	314 (27.2%)	183 (16.2%)	234 (13.2%)	230 (14.8%)
Americas	417 (7.4%)	0 (0.0%)	244 (21.7%)	169 (9.5%)	4 (0.3%)
Asia-Pacific	4,231 (75.4%)	842 (72.8%)	700 (62.1%)	1,374 (77.3%)	1,315 (84.9%)
G6PD status¶					
<30% activity	39 (0.8%)	24 (2.1%)	12 (1.8%)	3 (0.2%)	0 (0.0%)
≥30% activity	4,605 (99.2%)	1,119 (97.9%)	644 (98.2%)	1,595 (99.8%)	1,247 (100%)

G6PD – glucose-6-phosphate dehydrogenase; IQR – interquartile range; PQ – primaquine; Data recorded as number (%), median (IQR), mean (standard deviation) or n/N (%); Primaquine dosing, primaquine start day, primaquine duration, primaquine dose derivation, primaquine supervision and primaquine food administration are based on 4,453 patients administered primaquine; G6PD status was missing for 965 patients; *The nutritional status of children aged <5 years of age was calculated as a weight-for-age z-score, using the igrowup package developed by WHO,[38] with z-scores <-2 classified as having malnutrition. Malnutrition status was considered missing if Z scores were <-6 or >6. †If haemoglobin was not measured, haematocrit was converted to haemoglobin using the formula Haemoglobin (g/dL) = (Haematocrit (%) – 5.62) ÷ 2.60 (Lee *et al*, Malaria Journal 2008). ‡Relapse periodicity (the time between vivax relapses) was classified by geographic region as low and high, with high periodicity regions defined as having a median periodicity ≤47 days.[15] §Transmission intensity of study sites was classified as low (<1 case per 1,000 person years), moderate (1 to <10 cases per 1,000 person years) and high (≥10 cases per 1,000 person years) based on subnational malaria incidence estimates for the median year of enrolment.[1] ¶G6PD deficiency was categorised as deficient (<30% activity or an abnormal qualitative test) and normal (≥30% activity or a normal qualitative test).

Table S19. Number and percentage of patients reporting gastrointestinal symptoms on days 5-7

Symptom on any day between days 5-7	Children (<15 years)				Adults (≥15 years)			
	No primaquine	Low daily dose primaquine (<0.375 mg/kg/day)	Intermediate daily dose primaquine (≥0.375 & <0.75 mg/kg/day)	High daily dose primaquine (≥0.75 mg/kg/day)	No primaquine	Low daily dose primaquine (<0.375 mg/kg/day)	Intermediate daily dose primaquine (≥0.375 & <0.75 mg/kg/day)	High daily dose primaquine (≥0.75 mg/kg/day)
Any GI disturbance (Composite of 3)	11/388 (2.8%)	29/265 (10.9%)	40/428 (9.3%)	53/573 (9.2%)	18/505 (3.6%)	20/472 (4.2%)	47/695 (6.8%)	80/605 (13.2%)
Vomiting	2/388 (0.5%)	8/262 (3.1%)	8/404 (2.0%)	19/570 (3.3%)	0/505 (0.0%)	8/465 (1.7%)	9/632 (1.4%)	16/604 (2.6%)
Diarrhoea	2/388 (0.5%)	15/247 (6.1%)	9/422 (2.1%)	12/573 (2.1%)	2/501 (0.4%)	10/414 (2.4%)	10/653 (1.5%)	19/563 (3.4%)
Anorexia	8/182 (4.4%)	8/54 (14.8%)	28/388 (7.2%)	44/570 (7.7%)	18/253 (7.1%)	4/122 (3.3%)	34/588 (5.8%)	65/563 (11.5%)
Nausea					13/504 (2.6%)	21/386 (5.4%)	36/631 (5.7%)	56/604 (9.3%)
Abdominal pain					10/369 (2.7%)	21/269 (7.8%)	45/691 (6.5%)	102/605 (16.9%)
Dizziness					12/253 (4.7%)	9/86 (10.5%)	24/591 (4.1%)	33/563 (5.9%)

GI – gastrointestinal. Nausea, abdominal pain and dizziness were not assessed in children.

Figure S8. Association between primaquine daily dose and gastrointestinal symptoms on days 5-7

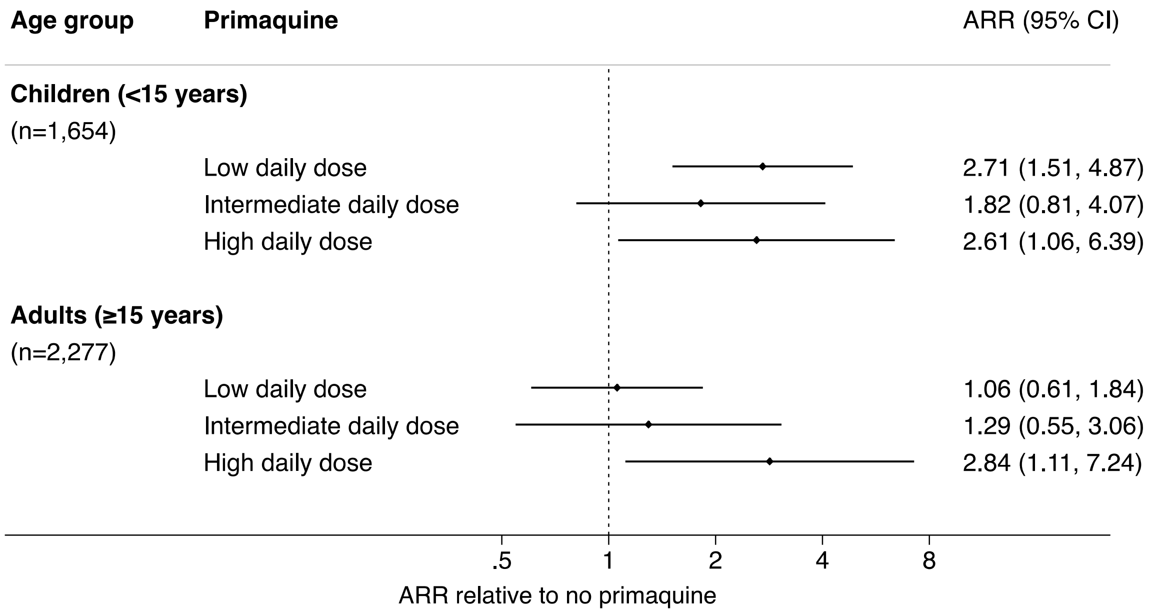

ARR – Adjusted Risk Ratio; CI – Confidence Interval; PQ – primaquine; Low daily dose PQ is a daily primaquine dose of <0.375 mg/kg/day; Intermediate daily dose PQ is a daily primaquine dose of 0.375 to <0.75 mg/kg/day; High daily dose PQ is a daily primaquine dose of ≥0.75 mg/kg/day; ARR – Adjusted Risk Ratio; Gastrointestinal symptoms are represented by a composite endpoint indicating presence of vomiting or diarrhoea or anorexia on days 5-7. Estimates are derived from a generalised estimating equation Poisson model with robust standard error estimates, adjusting for age category, sex and (log) baseline parasite density, with effect modification by age category and exchangeable correlation and clustering by study site, on data from 3,931 patients.

Figure S9. Covariate-adjusted estimated proportion of patients with gastrointestinal symptoms on days 5-7 by treatment regimen for all age categories

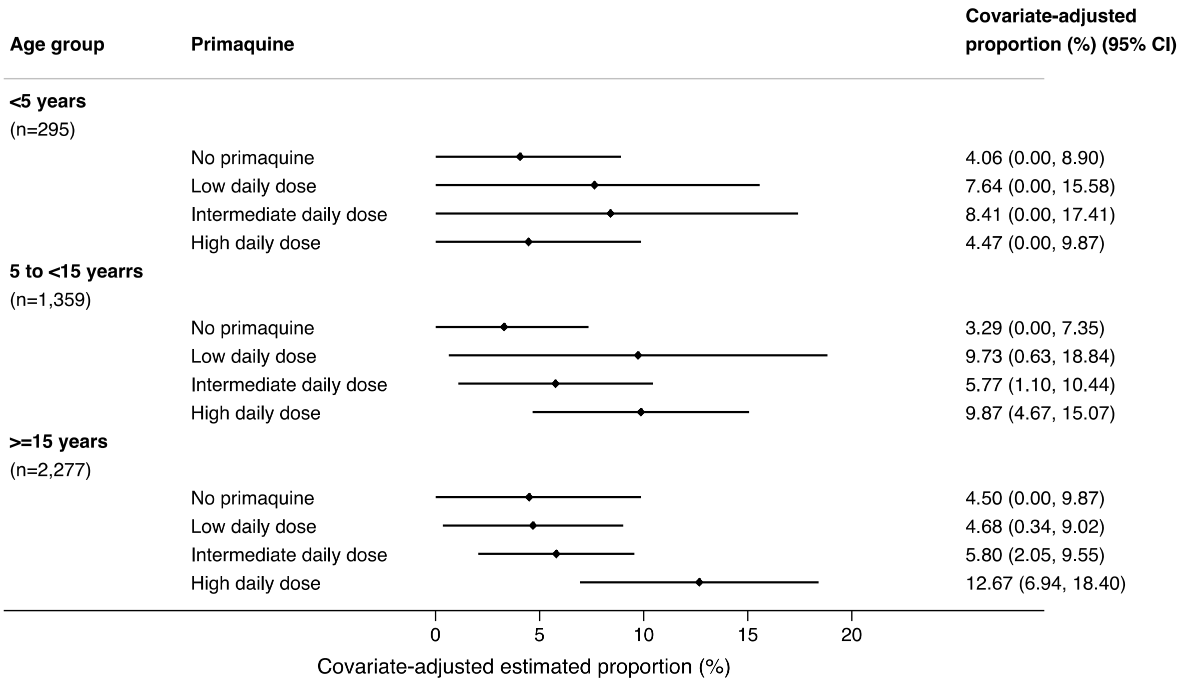

CI – Confidence Interval; PQ – primaquine; Low daily dose PQ is a daily primaquine dose of <0.375 mg/kg/day; Intermediate daily dose PQ is a daily primaquine dose of 0.375 to <0.75 mg/kg/day; High daily dose PQ is a daily primaquine dose of ≥0.75 mg/kg/day; Gastrointestinal symptoms are represented by a composite endpoint indicating presence of vomiting or diarrhoea or anorexia on days 5-7. The estimated proportion of patients with the composite gastrointestinal endpoint on days 5-7 was determined using a generalised estimating equation Poisson model with robust standard errors, adjusting for age category, sex and (log) baseline parasite density, with effect modification of treatment regimen by age category, and exchangeable correlation and clustering by study site. The model was fit using data from 3,931 patients and the adjusted proportions were estimated at mean values of the covariates.

Figure S10. Association between primaquine daily dose and gastrointestinal symptoms on days 5-7 for all age categories

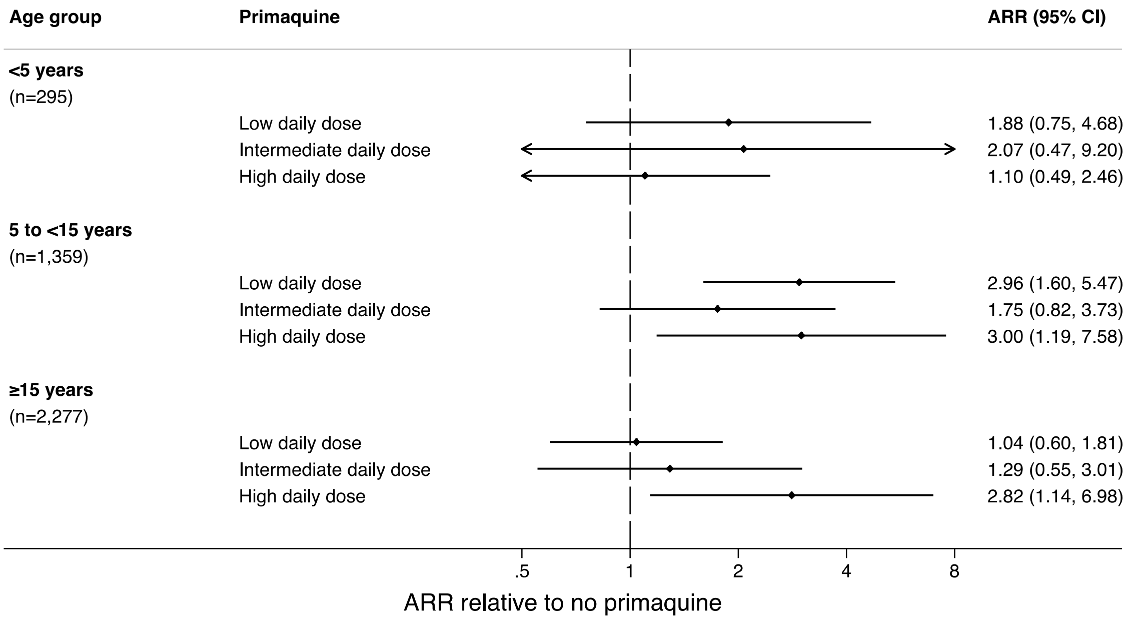

ARR – Adjusted Risk Ratio; CI – Confidence Interval; PQ – primaquine; Low daily dose PQ is a daily primaquine dose of <0.375 mg/kg/day; Intermediate daily dose PQ is a daily primaquine dose of 0.375 to <0.75 mg/kg/day; High daily dose PQ is a daily primaquine dose of ≥0.75 mg/kg/day; Gastrointestinal symptoms are represented by a composite endpoint indicating presence of vomiting or diarrhoea or anorexia on days 5-7. Estimates are derived from a generalised estimating equation Poisson model with robust standard errors, adjusting for age category, sex and (log) baseline parasite density, with effect modification of treatment regimen by age category, and exchangeable correlation and clustering by study site, on data from 3,931 patients.

Figure S11. Sensitivity analysis of the covariate-adjusted estimated proportion of patients with composite gastrointestinal outcome by treatment regimen on days 5-7

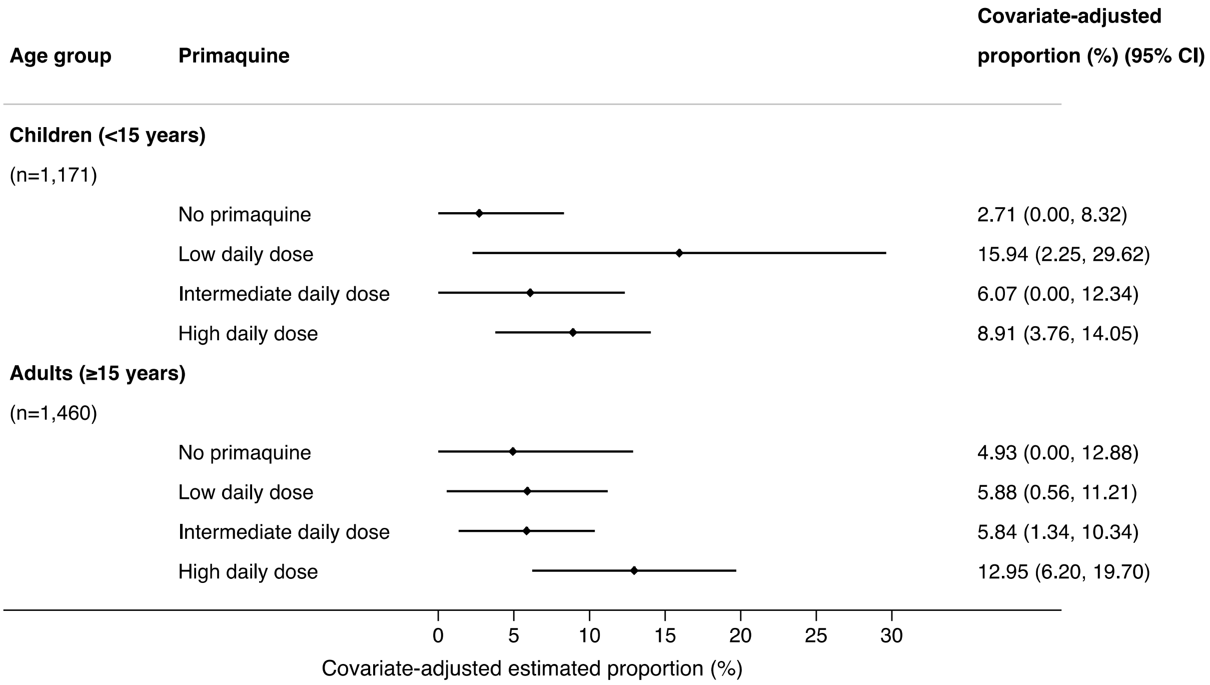

CI – Confidence Interval; PQ – primaquine; Low daily dose PQ is a daily primaquine dose of <0.375 mg/kg/day; Intermediate daily dose PQ is a daily primaquine dose of 0.375 to <0.75 mg/kg/day; High daily dose PQ is a daily primaquine dose of ≥0.75 mg/kg/day; Gastrointestinal symptoms are represented by a composite endpoint indicating presence of vomiting or diarrhoea or anorexia on days 5-7; Analysis was restricted to patients asked about all three of vomiting, diarrhoea and anorexia. The estimated proportion of patients with the composite gastrointestinal endpoint on days 5-7 was determined using a generalised estimating equation Poisson model with robust standard errors, adjusting for age category, sex and (log) baseline parasite density, with effect modification of treatment regimen by age category, and exchangeable correlation and clustering by study site. The model was fit using data from 2,631 patients, and the adjusted proportions were estimated at mean values of the covariates.

Figure S12. Sensitivity analysis of the relationship between the composite gastrointestinal outcome and primaquine daily dose on days 5-7

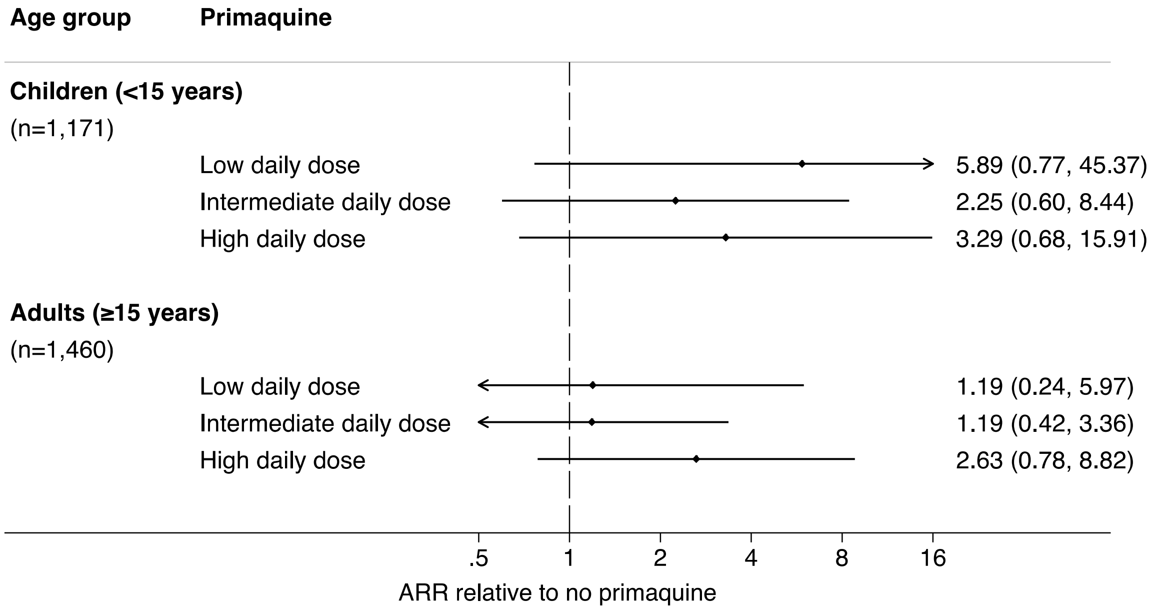

ARR – adjusted risk ratio; CI – confidence interval; PQ – primaquine; Low daily dose PQ is a daily primaquine dose of <0.375 mg/kg/day; Intermediate daily dose PQ is a daily primaquine dose of 0.375 to <0.75 mg/kg/day; High daily dose PQ is a daily primaquine dose of ≥0.75 mg/kg/day; Gastrointestinal symptoms are represented by a composite endpoint indicating presence of vomiting or diarrhoea or anorexia on days 5-7; Analysis restricted to patients asked about all three of vomiting, diarrhoea and anorexia. Estimates are derived from a generalised estimating equation Poisson model with robust standard errors, adjusting for age category, sex and (log) baseline parasite density, with effect modification of treatment by age category, and exchangeable correlation and clustering by study site, on data from 2,631 patients. Patients who did not receive primaquine served as the reference group.

Table S20. Number and percentage of patients reporting vomiting within an hour of a primaquine dose

	Children (<15 years)			Adults (≥15 years)		
	**Low daily dose PQ (<0.375 mg/kg/day)**	**Intermediate daily dose PQ (≥0.375 & <0.75 mg/kg/day)**	**High daily dose PQ (≥0.75 mg/kg/day)**	**Low daily dose PQ (<0.375 mg/kg/day)**	**Intermediate daily dose PQ (≥0.375 & <0.75 mg/kg/day)**	**High daily dose PQ (≥0.75 mg/kg/day)**
Vomiting within 1 hour of PQ on days 0-2	1/187 (0.5%)	14/561 (2.5%)	14/696 (2.0%)	8/355 (2.3%)	10/949 (1.1%)	7/807 (0.9%)
Vomiting within 1 hour of PQ on days 3-14	2/91 (2.2%)	2/537 (0.4%)	1/681 (0.1%)	1/125 (0.8%)	3/940 (0.3%)	3/790 (0.4%)

PQ - primaquine

Figure S13. Covariate-adjusted estimated proportion of patients with gastrointestinal symptoms on days 1-2 by age group and treatment arm

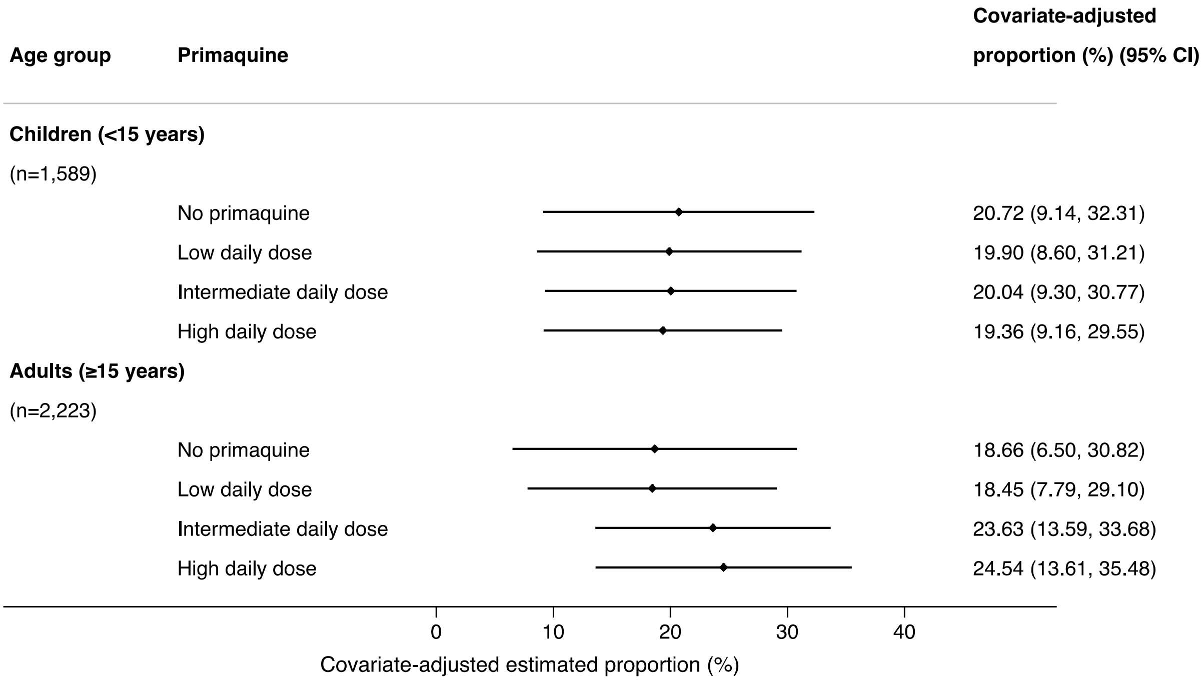

CI – confidence interval; PQ – primaquine; Low daily dose PQ is a daily primaquine dose of <0.375 mg/kg/day; Intermediate daily dose PQ is a daily primaquine dose of 0.375 to <0.75 mg/kg/day; High daily dose PQ is a daily primaquine dose of ≥0.75 mg/kg/day; The estimated proportion of patients with the composite gastrointestinal endpoint on days 1-2 was determined from a generalised estimating equation Poisson model with robust standard error estimates, adjusting for age category, sex and (log) baseline parasite density, with effect modification by age category, exchangeable correlation and clustering by study site. The model was fit to data from 3,812 patients and the adjusted proportions were estimated at mean values of the covariates.

Figure S14. Association between primaquine daily dose and gastrointestinal symptoms on days 1-2

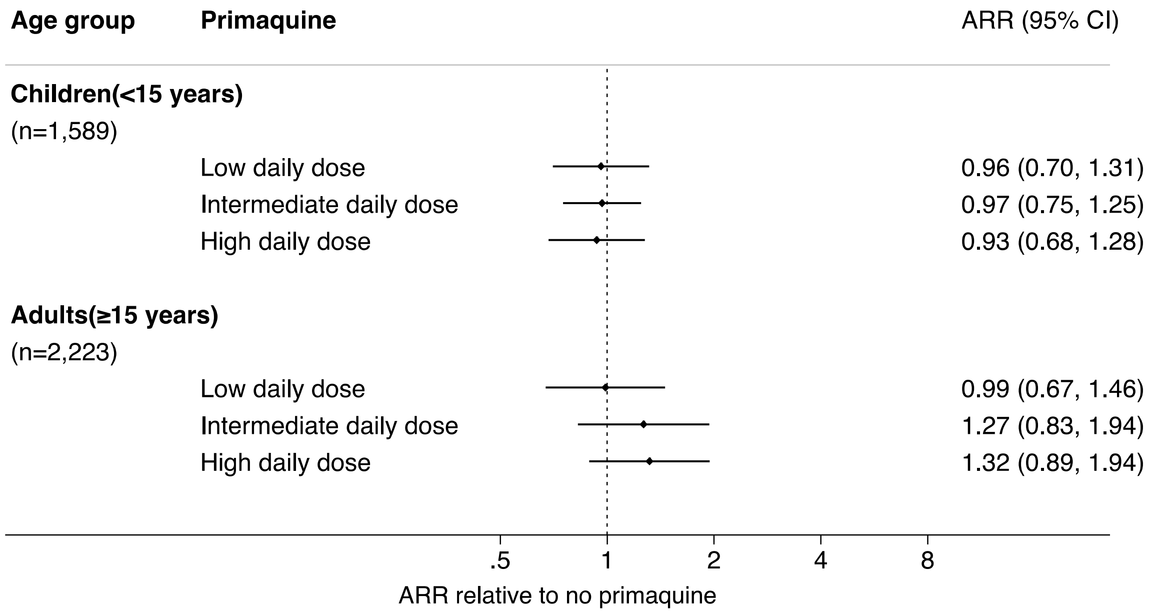

ARR – adjusted risk ratio; CI – Confidence Interval; PQ – primaquine; Low daily dose PQ is a daily primaquine dose of <0.375 mg/kg/day; Intermediate daily dose PQ is a daily primaquine dose of 0.375 to <0.75 mg/kg/day; High daily dose PQ is a daily primaquine dose of ≥0.75 mg/kg/day; ARR – Adjusted Risk Ratio; Association between primaquine daily dose and the composite gastrointestinal endpoint on days 1-2 was assessed by a generalised estimating equation Poisson model with robust standard error estimates, adjusting for age category, sex and (log) baseline parasite density, with effect modification by age category, exchangeable correlation and clustering by study site. The model was fit to data from 3,812 patients. Patients who did not receive primaquine served as the reference group.

References S1. Studies not included in analysis

56. Adak T, Valecha N, Sharma VP. Plasmodium vivax polymorphism in a clinical drug trial. *Clin Diagn Lab Immunol* 2001; **8**(5): 891-4.

57. Carmona-Fonseca J, Maestre A. Prevention of Plasmodium vivax malaria recurrence: efficacy of the standard total dose of primaquine administered over 3 days. *Acta Trop* 2009; **112**(2): 188-92.

58. Carmona-Fonseca J. Vivax malaria in children: Recurrences with standard total dose of primaquine administered in 3 vs. 7 days *Iatreia* 2010; **23**(1): 10-20.

59. Valecha N, Savargaonkar D, Srivastava B, et al. Comparison of the safety and efficacy of fixed-dose combination of arterolane maleate and piperaquine phosphate with chloroquine in acute, uncomplicated Plasmodium vivax malaria: a phase III, multicentric, open-label study. *Malar J* 2016; **15**: 42.

60. Dharmawardena P, Rodrigo C, Mendis K, et al. Response of imported malaria patients to antimalarial medicines in Sri Lanka following malaria elimination. *PLoS One* 2017; **12**(11): e0188613.

61. Poespoprodjo JR, Burdam FH, Candrawati F, et al. Supervised versus unsupervised primaquine radical cure for the treatment of falciparum and vivax malaria in Papua, Indonesia: a cluster-randomised, controlled, open-label superiority trial. *Lancet Infect Dis* 2022; **22**(3): 367-76.

62. Mekonnen DA, Abadura GS, Behaksra SW, et al. Treatment of uncomplicated Plasmodium vivax with chloroquine plus radical cure with primaquine without G6PDd testing is safe in Arba Minch, Ethiopia: assessment of clinical and parasitological response. *Malar J* 2023; **22**(1): 135.

63. Moore BR, Salman S, Tobe R, et al. Short-course, high-dose primaquine regimens for treatment of liver-stage vivax malaria in children. *Int J Infect Dis* 2023.

64. Sutanto I, Soebandrio A, Ekawati LL, et al. Tafenoquine co-administered with dihydroartemisinin-piperaquine for the radical cure of Plasmodium vivax malaria (INSPECTOR): a randomised, placebo-controlled, efficacy and safety study. *Lancet Infect Dis* 2023.

65. Woon SA, Moore BR, Laman M, et al. Ultra-short course, high-dose primaquine to prevent Plasmodium vivax infection following uncomplicated pediatric malaria: A randomized, open-label, non-inferiority trial of early versus delayed treatment. *Int J Infect Dis* 2023; **130**: 189-95.

66. Carmona-Fonseca J, Alvarez G. [Plasmodium vivax malaria: treatment of primary attacks with primaquine, in three different doses, and a fixed dose of chloroquine, Antioquia, Colombia, 2003-2004]. *Biomedica* 2006; **26**(3): 353-65.

67. Daneshvar C, Davis TM, Cox-Singh J, et al. Clinical and parasitological response to oral chloroquine and primaquine in uncomplicated human Plasmodium knowlesi infections. *Malar J* 2010; **9**: 238.

68. Saravu K, Acharya V, Kumar K, Kumar R. Plasmodium vivax remains responsive to chloroquine with primaquine treatment regimen: a prospective cohort study from tertiary care teaching hospital in southern India. *Trop Doct* 2012; **42**(3): 163-4.

69. Arcelia F, Pasaribu AP, Yanni GN. Effectiveness of dihydroartemisinin-piperaquine after 10 years as treatment for vivax malaria in Indonesia. *J Infect Dev Ctries* 2023; **17**(5): 700-6.

70. Bergonzoli G, Rivers Cuadra JC. [Therapeutic efficacy of different antimalarial regimens in the Costa Rica-Nicaragua border region]. *Rev Panam Salud Publica* 2000; **7**(6): 366-70.

71. Mohapatra MK, Padhiary KN, Mishra DP, Sethy G. Atypical manifestations of Plasmodium vivax malaria. *Indian J Malariol* 2002; **39**(1-2): 18-25.

72. da Silva Rdo S, Pinto AY, Calvosa VS, de Souza JM. [Short course schemes for vivax malaria treatment]. *Rev Soc Bras Med Trop* 2003; **36**(2): 235-9.

73. Walsh DS, Wilairatana P, Tang DB, et al. Randomized trial of 3-dose regimens of tafenoquine (WR238605) versus low-dose primaquine for preventing Plasmodium vivax malaria relapse. *Clin Infect Dis* 2004; **39**(8): 1095-103.

74. Villalobos-Salcedo JM, Tada MS, Kimura E, Menezes MJ, Pereira da Silva LH. In-vivo sensitivity of Plasmodium vivax isolates from Rond nia (western Amazon region, Brazil) to regimens including chloroquine and primaquine. *Ann Trop Med Parasitol* 2000; **94**(8): 749-58.

75. Dunne MW, Singh N, Shukla M, et al. A double-blind, randomized study of azithromycin compared to chloroquine for the treatment of Plasmodium vivax malaria in India. *Am J Trop Med Hyg* 2005; **73**(6): 1108-11.

76. Lee SW, Lee M, Lee DD, et al. Biological resistance of hydroxychloroquine for Plasmodium vivax malaria in the Republic of Korea. *Am J Trop Med Hyg* 2009; **81**(4): 600-4.

77. Eibach D, Ceron N, Krishnalall K, et al. Therapeutic efficacy of artemether-lumefantrine for Plasmodium vivax infections in a prospective study in Guyana. *Malar J* 2012; **11**: 347.

78. Orjuela-Sanchez P, da Silva NS, da Silva-Nunes M, Ferreira MU. Recurrent parasitemias and population dynamics of Plasmodium vivax polymorphisms in rural Amazonia. *Am J Trop Med Hyg* 2009; **81**(6): 961-8.

79. Alvarez G, Pineros JG, Tobon A, et al. Efficacy of three chloroquine-primaquine regimens for treatment of Plasmodium vivax malaria in Colombia. *Am J Trop Med Hyg* 2006; **75**(4): 605-9.

80. Leslie T, Rab MA, Ahmadzai H, et al. Compliance with 14-day primaquine therapy for radical cure of vivax malaria--a randomized placebo-controlled trial comparing unsupervised with supervised treatment. *Trans R Soc Trop Med Hyg* 2004; **98**(3): 168-73.

81. Leslie T, Mayan I, Mohammed N, et al. A randomised trial of an eight-week, once weekly primaquine regimen to prevent relapse of plasmodium vivax in Northwest Frontier Province, Pakistan. *PLoS One* 2008; **3**(8): e2861.

82. Abdallah TM, Ali AA, Bakri M, Gasim GI, Musa IR, Adam I. Efficacy of artemether-lumefantrine as a treatment for uncomplicated Plasmodium vivax malaria in eastern Sudan. *Malar J* 2012; **11**: 404.

83. Marques MM, Costa MR, Santana Filho FS, et al. Plasmodium vivax chloroquine resistance and anemia in the western Brazilian Amazon. *Antimicrob Agents Chemother* 2014; **58**(1): 342-7.

84. Gomes Mdo S, Vieira JL, Machado RL, et al. Efficacy in the treatment of malaria by Plasmodium vivax in Oiapoque, Brazil, on the border with French Guiana: the importance of control over external factors. *Malar J* 2015; **14**: 402.

85. Rishikesh K, Kamath A, Hande MH, et al. Therapeutic assessment of chloroquine-primaquine combined regimen in adult cohort of Plasmodium vivax malaria from a tertiary care hospital in southwestern India. *Malar J* 2015; **14**: 310.

86. Ley B, Alam MS, Thriemer K, et al. G6PD Deficiency and Antimalarial Efficacy for Uncomplicated Malaria in Bangladesh: A Prospective Observational Study. *PLoS One* 2016; **11**(4): e0154015.

87. Saravu K, Kumar R, Ashok H, et al. Therapeutic Assessment of Chloroquine-Primaquine Combined Regimen in Adult Cohort of Plasmodium vivax Malaria from Primary Care Centres in Southwestern India. *PLoS One* 2016; **11**(6): e0157666.

88. Brasil LW, Rodrigues-Soares F, Santoro AB, et al. CYP2D6 activity and the risk of recurrence of Plasmodium vivax malaria in the Brazilian Amazon: a prospective cohort study. *Malar J* 2018; **17**(1): 57.

89. Xu S, Zeng W, Ngassa Mbenda HG, et al. Efficacy of directly-observed chloroquine-primaquine treatment for uncomplicated acute Plasmodium vivax malaria in northeast Myanmar: A prospective open-label efficacy trial. *Travel Med Infect Dis* 2020; **36**: 101499.

90. Van den Eede P, Soto-Calle VE, Delgado C, et al. Plasmodium vivax sub-patent infections after radical treatment are common in Peruvian patients: results of a 1-year prospective cohort study. *PLoS One* 2011; **6**(1): e16257.

91. Negreiros S, Farias S, Viana GM, et al. Efficacy of Chloroquine and Primaquine for the Treatment of Uncomplicated Plasmodium vivax Malaria in Cruzeiro do Sul, Brazil. *Am J Trop Med Hyg* 2016; **95**(5): 1061-8.

92. Fukuda MM, Krudsood S, Mohamed K, et al. A randomized, double-blind, active-control trial to evaluate the efficacy and safety of a three day course of tafenoquine monotherapy for the treatment of Plasmodium vivax malaria. *PLoS One* 2017; **12**(11): e0187376.

93. Pham TV, Nguyen HV, Aguirre AR, et al. Plasmodium vivax morbidity after radical cure: A cohort study in Central Vietnam. *PLoS Med* 2019; **16**(5): e1002784.

94. Abdon NP, Pinto AY, das Silva Rdo S, de Souza JM. [Assessment of the response to reduced treatment schemes for vivax malaria]. *Rev Soc Bras Med Trop* 2001; **34**(4): 343-8.

95. Dua VK, Sharma VP. Plasmodium vivax relapses after 5 days of primaquine treatment, in some industrial complexes of India. *Ann Trop Med Parasitol* 2001; **95**(7): 655-9.

96. Duarte EC, Pang LW, Ribeiro LC, Fontes CJ. Association of subtherapeutic dosages of a standard drug regimen with failures in preventing relapses of vivax malaria. *Am J Trop Med Hyg* 2001; **65**(5): 471-6.

97. Solari Soto L, Soto Tarazona AR, Mendoza Requena D, Llanos Cuentas EA. Ensayo clinico del tratamiento de la malaria vivax con esquema acortado de primaquina comparado con el esquema tradicional. *Rev Soc Peru Med Interna* 2002; **15**(4): 197-9.

98. Yadav RS, Ghosh SK. Radical curative efficacy of five-day regimen of primaquine for treatment of Plasmodium vivax malaria in India. *J Parasitol* 2002; **88**(5): 1042-4.

99. Fernandopulle BM, Weeraratne CL, Weerasuriya K, Karunaweera ND. Efficacy of a five-day course of primaquine in preventing relapses in Plasmodium vivax malaria--a pilot study. *Ceylon Med J* 2003; **48**(1): 32.

100. Rajgor DD, Gogtay NJ, Kadam VS, et al. Efficacy of a 14-day primaquine regimen in preventing relapses in patients with Plasmodium vivax malaria in Mumbai, India. *Trans R Soc Trop Med Hyg* 2003; **97**(4): 438-40.

101. Takeuchi R, Lawpoolsri S, Imwong M, et al. Directly-observed therapy (DOT) for the radical 14-day primaquine treatment of Plasmodium vivax malaria on the Thai-Myanmar border. *Malar J* 2010; **9**: 308.

102. Maneeboonyang W, Lawpoolsri S, Puangsa-Art S, et al. Directly observed therapy with primaquine to reduce the recurrence rate of plasmodium vivax infection along the Thai-Myanmar border. *Southeast Asian J Trop Med Public Health* 2011; **42**(1): 9-18.

103. Muhamad P, Ruengweerayut R, Chacharoenkul W, Rungsihirunrat K, Na-Bangchang K. Monitoring of clinical efficacy and in vitro sensitivity of Plasmodium vivax to chloroquine in area along Thai Myanmar border during 2009-2010. *Malar J* 2011; **10**: 44.

104. Graf PC, Durand S, Alvarez Antonio C, et al. Failure of Supervised Chloroquine and Primaquine Regimen for the Treatment of Plasmodium vivax in the Peruvian Amazon. *Malar Res Treat* 2012; **2012**: 936067.

105. Ganguly S, Saha P, Guha SK, et al. In vivo therapeutic efficacy of chloroquine alone or in combination with primaquine against vivax malaria in Kolkata, West Bengal, India, and polymorphism in pvmdr1 and pvcrt-o genes. *Antimicrob Agents Chemother* 2013; **57**(3): 1246-51.

106. Liu H, Yang HL, Xu JW, Wang JZ, Nie RH, Li CF. Artemisinin-naphthoquine combination versus chloroquine-primaquine to treat vivax malaria: an open-label randomized and non-inferiority trial in Yunnan Province, China. *Malar J* 2013; **12**: 409.

107. Macareo L, Lwin KM, Cheah PY, Yuentrakul P, Miller RS, Nosten F. Triangular test design to evaluate tinidazole in the prevention of Plasmodium vivax relapse. *Malar J* 2013; **12**: 173.

108. Delgado-Ratto C, Soto-Calle VE, Van den Eede P, et al. Population structure and spatio-temporal transmission dynamics of Plasmodium vivax after radical cure treatment in a rural village of the Peruvian Amazon. *Malar J* 2014; **13**: 8.

109. Cheoymang A, Ruenweerayut R, Muhamad P, Rungsihirunrat K, Na-Bangchang K. Patients' adherence and clinical effectiveness of a 14-day course of primaquine when given with a 3-day chloroquine in patients with Plasmodium vivax at the Thai-Myanmar border. *Acta Trop* 2015; **152**: 151-6.

67/110. Pareek A, Chandurkar N, Gogtay N, et al. Sustained Release Formulation of Primaquine for Prevention of Relapse of Plasmodium vivax Malaria: A Randomized, Double-Blind, Comparative, Multicentric Study. *Malar Res Treat* 2015; **2015**: 579864.

111. Mac Donald-Ottevanger MS, Adhin MR, Jitan JK, Bretas G, Vreden SG. Primaquine double dose for 7 days is inferior to single-dose treatment for 14 days in preventing Plasmodium vivax recurrent episodes in Suriname. *Infect Drug Resist* 2018; **11**: 3-8.

112. Pukrittayakamee S, Chantra A, Simpson JA, et al. Therapeutic responses to different antimalarial drugs in vivax malaria. *Antimicrob Agents Chemother* 2000; **44**(6): 1680-5.

113. Singh RK. Emergence of chloroquine-resistant vivax malaria in south Bihar (India). *Trans R Soc Trop Med Hyg* 2000; **94**(3): 327.

114. Buchachart K, Krudsood S, Singhasivanon P, et al. Effect of primaquine standard dose (15 mg/day for 14 days) in the treatment of vivax malaria patients in Thailand. *Southeast Asian J Trop Med Public Health* 2001; **32**(4): 720-6.

115. Congpuong K, Na-Bangchang K, Thimasarn K, Tasanor U, Wernsdorfer WH. Sensitivity of Plasmodium vivax to chloroquine in Sa Kaeo Province, Thailand. *Acta Trop* 2002; **83**(2): 117-21.

116. Machado RL, de Figuereido Filho AF, Calvosa VS, Figueredo MC, Nascimento JM, Povoa MM. Correlation between Plasmodium vivax variants in Belem, Para State, Brazil and symptoms and clearance of parasitaemia. *Braz J Infect Dis* 2003; **7**(3): 175-7.

117. Pinto AY, Azevedo CH, da Silva JB, de Souza JM. Assessment of chloroquine single dose treatment of malaria due to Plasmodium vivax in Brazilian Amazon. *Rev Inst Med Trop Sao Paulo* 2003; **45**(6): 327-31.

118. Hapuarachchi HA, Dayanath MY, Abeysundara S, Bandara KB, Abeyewickreme W, de Silva NR. Chloroquine resistant falciparum malaria among security forces personnel in the Northern Province of Sri Lanka. *Ceylon Med J* 2004; **49**(2): 47-51.

119. Krudsood S, Tangpukdee N, Muangnoicharoen S, et al. Clinical efficacy of chloroquine versus artemether-lumefantrine for Plasmodium vivax treatment in Thailand. *Korean J Parasitol* 2007; **45**(2): 111-4.

120. Krudsood S, Wilairatana P, Tangpukdee N, et al. Safety and tolerability of elubaquine (bulaquine, CDRI 80/53) for treatment of Plasmidium vivax malaria in Thailand. *Korean J Parasitol* 2006; **44**(3): 221-8.

121. Tasanor O, Ruengweerayut R, Sirichaisinthop J, Congpuong K, Wernsdorfer WH, Na-Bangchang K. Clinical-parasitological response and in-vitro sensitivity of Plasmodium vivax to chloroquine and quinine on the western border of Thailand. *Trans R Soc Trop Med Hyg* 2006; **100**(5): 410-8.

122. Maguire JD, Krisin, Marwoto H, Richie TL, Fryauff DJ, Baird JK. Mefloquine is highly efficacious against chloroquine-resistant Plasmodium vivax malaria and Plasmodium falciparum malaria in Papua, Indonesia. *Clin Infect Dis* 2006; **42**(8): 1067-72.

123. Nateghpour M, Edrissian G, Torabi A, et al. Monitoring of Plasmodium vivax and Plasmodium falciparum response to chloroquine in Bandar-Abbas district, Hormozgan province, Iran. [Arabic]. *Tehran Uni Med J* 2009; **67**(3): 178-83.

124. Kim YK, Kim C, Park I, Kim HY, Choi JY, Kim JM. Therapeutic efficacy of chloroquine in Plasmodium vivax and the pvmdr1 polymorphisms in the Republic of Korea under mass chemoprophylaxis. *Am J Trop Med Hyg* 2011; **84**(4): 532-4.

125. Pedro RS, Guaraldo L, Campos DP, Costa AP, Daniel-Ribeiro CT, Brasil P. Plasmodium vivax malaria relapses at a travel medicine centre in Rio de Janeiro, a non-endemic area in Brazil. *Malar J* 2012; **11**: 245.

126. Zhu G, Lu F, Cao J, et al. Blood stage of Plasmodium vivax in central China is still susceptible to chloroquine plus primaquine combination therapy. *Am J Trop Med Hyg* 2013; **89**(1): 184-7.

127. Azarian Moghadam HN, Raeisi M, Motevalli Haghi A, Edrissian A, Farivar G. Monitoring the Response of *Plasmodium vivax* to Chloroquine and Uncomplicated *P. falciparum* to Artesunate-fansidar Antimalarials in Southeastern Iran. *Iran J Parasitol* 2018; **13**(1): 31-8.

128. Mesa-Echeverry E, Niebles-Bolivar M, Tobon-Castano A. Chloroquine-Primaquine Therapeutic Efficacy, Safety, and Plasma Levels in Patients with Uncomplicated Plasmodium vivax Malaria in a Colombian Pacific Region. *Am J Trop Med Hyg* 2019; **100**(1): 72-7.
